# Nanoparticle-Enabled Plasma Proteomics of Experimental Atherosclerosis in Mice

**DOI:** 10.1016/j.jacbts.2026.101649

**Published:** 2026-08-19

**Authors:** Constance Delwarde, Joan T. Matamalas, Sarvesh Chelvanambi, Taku Kasai, Gabriel Shlayen, Diego V. Santinelli-Pestana, Maedeh Zamani, Elena Aikawa, Masanori Aikawa, Sasha A. Singh

**Affiliations:** a Center for Interdisciplinary Cardiovascular Sciences, Department of Medicine, Brigham and Women's Hospital, Harvard Medical School, Boston, Massachusetts, USA; b Seer Inc, Redwood City, California, USA; c Center for Excellence in Vascular Biology, Department of Medicine, Brigham and Women's Hospital, Harvard Medical School, Boston, Massachusetts, USA; d Channing Division of Network Medicine, Department of Medicine, Brigham and Women's Hospital and Harvard Medical School, Boston, Massachusetts, USA

**Keywords:** coronary heart disease, mass spectrometry, preclinical model, protein coronas

## Abstract

Dyslipidemia, marked by elevated low-density lipoprotein cholesterol, is a major risk factor for coronary heart disease. Mouse experimental systems, such as *Ldlr*^*−/−*^ mice that develop atherosclerosis and metabolic disorders when fed a high-fat diet (HFD), are crucial for studying disease mechanisms and identifying potential biomarkers. Using recent nanoparticle-dependent enrichment of plasma proteins and mass spectrometry, we quantified 5,080 plasma proteins from *Ldlr*^*−/−*^ mice fed a chow or HFD for 3 or 6 months. Proteins with sustained enrichment in HFD were indicative of liver cell subtypes, whereas proteins that increased from 3 to 6 months were indicative of inflammation and endothelial activation. Proteins/genes from human cardiovascular genomic and proteomic studies were also detected in the plasma and aorta including MMP12, LPL, and HP that increased with HFD. These data provide many avenues for understanding disease pathology and uncovering novel molecular contributors to cardiovascular diseases.

## Highlights

• Circulating proteins reflect disease mechanisms and hold biomarker potential, yet the plasma proteome of experimental atherosclerosis in the widely used *Ldlr*^*−/−*^ mice has never been systemically profiled.
• Using state-of-the-art protein coronas forming and mass spectrometry technologies, we enriched and identified 5,000+ plasma proteins from *Ldlr*^*−/−*^ mice on a high-fat diet.
• The protein signatures, spanning dyslipidemia, inflammation, and endothelial activation, align with previous mouse and human studies, delivering a novel resource for biomarker and therapeutic discovery.

## Visual Abstract

**Figure:**
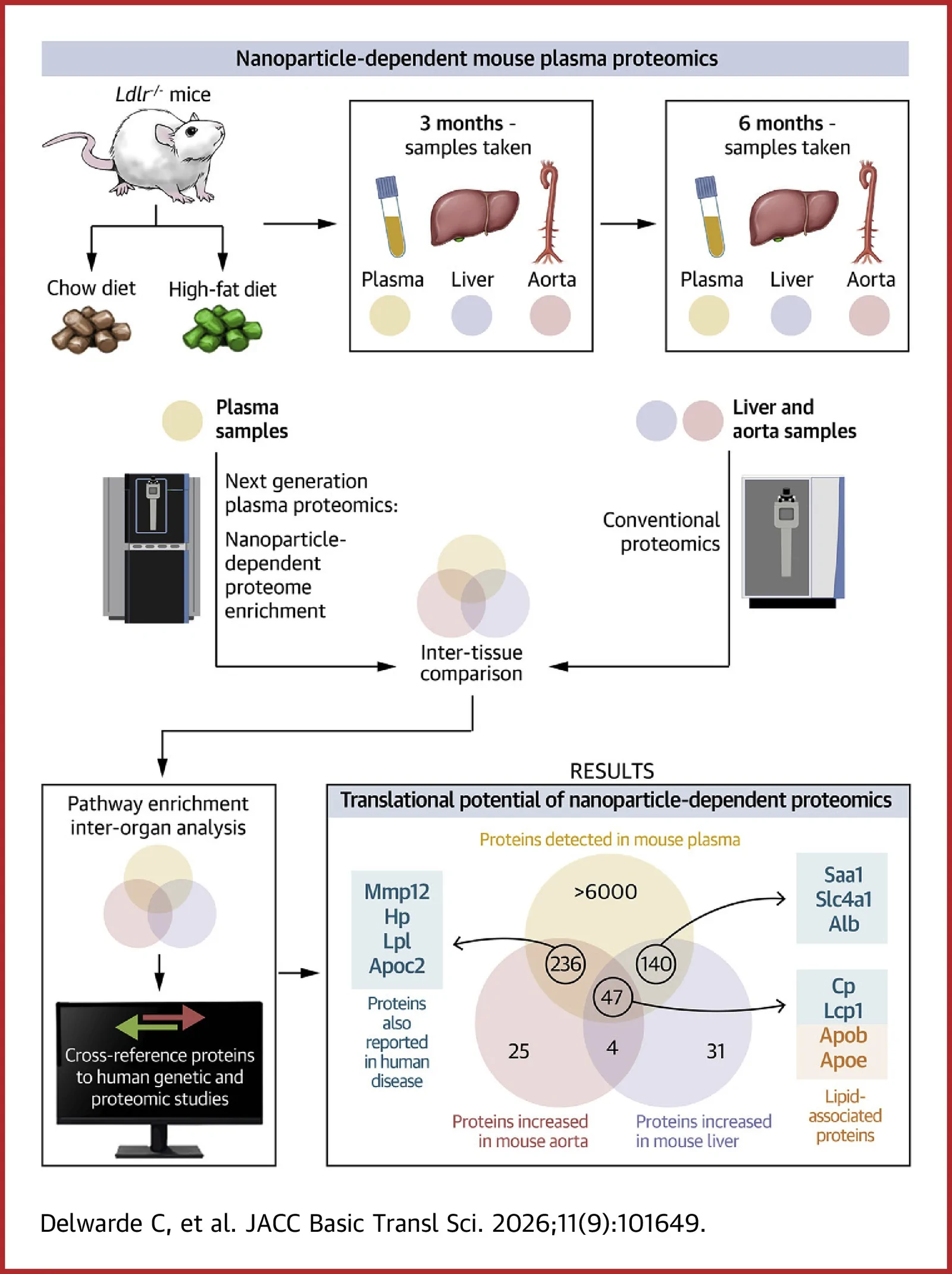

Atherosclerosis remains one of the leading causes of morbidity and mortality worldwide, contributing significantly to the global burden of cardiovascular disease.^1^ The pathogenesis of atherosclerotic vascular disease is complex and multifactorial, involving lipid metabolism disturbances and chronic inflammation.

Dyslipidemia promotes atherosclerosis primarily by elevated plasma levels of low-density lipoprotein cholesterol.^2^, ^3^, ^4^, ^5^, ^6^ Atherosclerosis is a chronic disease whose progression cannot be easily studied in humans; therefore, experimental systems, particularly in mice, are pivotal for research.^7^, ^8^, ^9^ The two most common approaches for in vivo atherosclerosis studies rely on genetic deletion of either *Apoe* or *Lldr*.^10^, ^11^, ^12^ The smaller high-density lipoproteins (HDLs) usually predominate the plasma of wild-type mice, but in *Apoe*^*−/−*^ and *Ldlr*^*−/−*^ mice, plasma lipoprotein species shift towards the APOB100/48 particles very–low density lipoprotein (VLDL), intermediate-density lipoprotein (IDL), and low-density lipoprotein (LDL). *Apoe*^*−/−*^ mice accumulate chylomicron and VLDL remnants, whereas *Ldlr*^*−/−*^ mice accumulate VLDL and LDL in a high-cholesterol, high-fat diet (HFD).^10^, ^11^, ^12^, ^13^, ^14^ LDL deposition into the subendothelial space is one of the earliest events in atherosclerosis, which precedes immune responses, such as macrophage accumulation in humans.^15^ LDL's retention within the intima is facilitated by binding to extracellular matrix (ECM) components such as proteoglycans and collagen.^15^^,^^16^

To identify candidate disease drivers of atherosclerosis in the *Ldlr*^*−/−*^ model, molecular profiling workflows such as proteomics have examined solid organs including the aorta and liver.^9^^,^^17^, ^18^, ^19^, ^20^ Such studies aim to characterize disease progression driven by local and remote (organ-to-organ crosstalk) processes.^9^^,^^17^ Although circulating proteins are expected to reflect key disease mechanisms and serve as potential biomarkers, the murine plasma proteome has remained an underexplored resource in cardiovascular disease research. Plasma/serum is dominated by approximately 300 proteins, including albumin, apolipoproteins, immunoglobulins, fibrinogens, and complement factors, that mask the presence of potential novel disease–informative biomarkers that span at least a 10^10^-fold dynamic range.^21^^,^^22^ This masking phenomenon has been particularly challenging for mass spectrometry–dependent profiling. However, multiple gains have been made on the human plasma front, primarily to leverage the common practice of acquiring body fluids to monitor biomarkers with prognostic or diagnostic value. These and other clinical applications have sustained interest in innovative deep plasma proteome profiling.^22^ Affinity binding-dependent workflows (using aptamers or antibodies) emerged over a decade ago. They rely on an amplicon or fluorophore readout for protein detection.^23^ These approaches provide panels surpassing 4,000 target human proteins, but limitations of these technologies exist, including the lack of nonhuman panels, which limits their applicability in animal studies.^23^

Recent advances in nanoparticle-dependent protein corona-forming workflows have made mass spectrometry plasma proteomics increasingly feasible.^24^ The engineered surfaces of these nanoparticles bind proteins in 2 phases—first nonspecifically by high-abundant proteins, followed by high-affinity lower-abundant proteins that displace the former at the surfaces of the nanoparticles; a process first described by Vroman and colleagues.^25^ The nanoparticle enrichment method therefore binds proteins based on chemistry rather than abundance,^26^ thereby enriching lower abundant proteins and compressing the dynamic range of protein signal for the mass spectrometer. Moreover, mass spectrometers have become increasingly faster, achieving more than 5,000 proteins within a 30-minute runtime, which is markedly quicker than conventional times of 60 minutes or more to yield comparable results. These platforms provide researchers with the means to implement mass spectrometry–based workflows to profile hundreds of preclinical or clinical plasma samples in a matter of days.^27^ We therefore took advantage of a nanoparticle-enrichment strategy previously applied to human plasma,^28^^,^^29^ to profile the plasma proteome of *Ldlr*^*−/−*^ mice fed a HFD or chow diet for 3 or 6 months, time points during which plaques form and become progressively diseased.^14^^,^^19^^,^^30^^,^^31^

## Methods

### Animal experiments

All animal procedures were approved and performed in compliance with Beth Israel Deaconess Medical Center's Institutional Animal Care and Use Committee (IACUC protocol #003-2020). Eight-week-old male *Ldlr*^*−/−*^ mice were acquired from the Jackson Laboratory (RRID:IMSR JAX:002,207). Upon arrival, the mice immediately began either a chow/control diet (LabDiet, catalog #5008i) or HFDs (Clinton/Cybulsky High Fat Rodent Diet [1.25% cholesterol], gamma-irradiation; Research Diets Inc, catalog #D12108 Ci) for 3 (n = 27) or 6 (n = 12) months.^9^

### Plasma sample preparation with the Proteograph XT Assay protocol

Mice were euthanized using the CO_2_ chamber. Then, blood was collected from the left ventricle.^9^^,^^32^ Plasma samples were processed with Proteograph Product Suite using the Proteograph XT Assay,^33^ using 75 μL of each plasma sample that was mixed with 165 μL of Reconstitution Buffer A (S55R3090) to yield a final volume of 240 μL and were then transferred to Seer Sample Tubes for automated processing with the Proteograph XT Assay kit (S55R1100). Plasma samples were aliquoted into 2 wells of a Sample Prep Plate, and proteins were quantitatively captured in protein coronas associated with nanoparticle suspensions A and B. Proteins were subsequently denatured, reduced, alkylated, and subjected to proteolytic digestion (trypsin and Lys-C). Peptides were purified and yields were determined (Thermo Fisher Scientific, catalog #23290). Peptides were dried down overnight with a vacuum concentrator and reconstituted with a reconstitution buffer to a concentration of 50 ng/μL. All plasma samples in the study were processed across 2 Proteograph XT Assay plates.

### Plasma sample preparation with the direct digestion method

For direct digestion of 5 μL diluted neat plasma samples, proteins were denatured, reduced, alkylated, and subjected to proteolytic digestion (Trypsin and Lys-C) for 3 hours at 37 °C. Peptides were purified by solid phase extraction, and yields were determined (Thermo Fisher Scientific catalog #23290).

### Data-independent acquisition mass spectrometry

For data-independent acquisition (DIA), 8 μL of reconstituted peptide mixture from each nanoparticle preparation was analyzed, resulting in a constant 400-ng load on column for nanoparticle A and nanoparticle B samples. Each sample was analyzed with a Vanquish NEO nanoLC system coupled with an Orbitrap Astral mass spectrometer (Thermo Fisher Scientific) using a trap-and-elute configuration. First, the peptides were loaded onto an Acclaim PepMap 100 C18 (0.3 mm ID × mm) trap column and then separated on a Gen1 50 cm μPAC analytical column (Thermo Fisher Scientific) at a flow rate of 1 μL/min using a gradient of 4% to 35% mobile phase B (acetonitrile/0.1% formic acid) mixed into mobile phase A (water/0.1% formic acid) over 21 minutes, resulting in a 26-minute run time. The mass spectrometer was operated in DIA mode. MS1 scans were performed in the Orbitrap detector at 240,000 resolution, every 0.6 seconds with a 5-ms ion injection time or 500% automatic gain control (AGC; 500,000 ion) target. For DIA experiment, 200 fixed DIA window covering a 380 to 980 Th mass range were collected at the Astral detector per cycle with 3-Th precursor isolation windows, 25% normalized collision energy, and 5-ms ion injection times with a 500% (50,000 ions) active gain control maximum. MS2 scans were collected from m/z 150 to 2000. Window placement optimization was turned on. A source voltage of 1,500 V and an ion transfer tube temperature of 300 °C were used for all experiments.

### Tandem mass spectrometry spectral annotation for the plasma proteomes

DIA data were processed using Proteograph Analysis Suite (PAS). The spectra were processed using the DIA-NN search engine^34^ (version 1.8.1) in library-free mode searching tandem mass spectrometry (MS/MS) spectra against an in silico generated spectral library based on the Uniprot mouse database (UP000000589_10090), excluding fragment sequences, resulting in 46,734 mouse entries and an additional 91 contaminant entries. Library-free search parameters include trypsin protease, 1 missed cleavage, N-terminal methionine excision, fixed modification of cysteine carbamidomethylation, peptide length of 7 to 30 amino acids, precursor range of m/z 300 to 1800, and fragment ion range of m/z 200 to 1800. MS1 and MS2 mass accuracy was set to 3 and 8 ppm, respectively. Precursor and Protein Group false-discovery rate (FDR) thresholds were set at 1%. Quantification was performed on summed abundances of all unique peptides considering only precursors passing the FDR thresholds. PAS summarizes all nanoparticle values for a single protein into a single quantitative value. Specifically, a single protein may have been measured 2 times, once for each nanoparticle. To derive the single measurement value, PAS uses a maximum representation approach, whereby the single quantification value for a particular peptide or protein group represents the quantitation value of the nanoparticle most frequently measured across all samples.^35^

### Liver and aorta homogenization

The protocol was based on our previously published methods.^9^^,^^36^^,^^37^ These tissue homogenates were prepared for the present study as well as for our previous study investigating the corresponding ADP-ribosylome.^9^ For ADP-ribosylome yield purposes, before the standard homogenization step, 10 frozen aortas were combined and minced for a total of 4 sets of 10 pooled aortas per diet and time point (ie, n = 40 aortas per diet and time point). An individual liver was sufficient to extract the proteome and ADP-ribosylome (n = 6 livers per time point).^9^ Each liver or aorta pool was transferred to a Precellys 2 mL tube from the Soft Tissue Homogenizing Ceramic Beads Kit (Bertin Technologies, catalog #CK14) for liver, and from the Hard Tissue Homogenizing Ceramic Beads kit (Bertin Technologies, t catalog #CK28) for aorta. Then, the following protocol was applied to both aorta and liver samples. A modified RIPA buffer (0.5 mL, 50 mM Tris-HCl pH 7.4 [Boston BioProducts, catalog #BM-327], 0.4 M NaCl [Sigma-Aldrich, catalog #S9888], 1.0 mM EDTA [Boston Bio Products, catalog t#BM-150], 1.0% nonidet P-40 [Sigma-Aldrich, catalog #74385], 0.1% sodium deoxycholate [Sigma-Aldrich, catalog #D6750], 40 μM PJ34, 1.0 μM ADP-HPD, protease inhibitor cocktail [Sigma-Aldrich, catalog #P8340], and phosphatase inhibitor [Sigma-Aldrich, catalog #4906845001]) was added to tubes for protein analysis. Tissues were homogenized in a Precellys 24 Tissue Homogenizer (Bertin Technologies, catalog #P000669-PR240-A) using three 10-second cycles at 5,000 rpm that were then cooled on ice for 15 minutes. Tissue debris was then removed by centrifugation (3,000 rpm, 4 °C, 5 minutes).

### Liver and aorta proteolysis steps

For protein precipitation, 5× volume of acetone (Fisher Scientific, catalog #A949-1) was added to the tissue homogenates^9^^,^^38^^,^^39^ and then resuspended in a denaturation buffer (6.0 M urea [Sigma-Aldrich, catalog #U4884], 2.0 M thiourea [Sigma-Aldrich, catalog #T7875], 10 mM HEPES [Boston BioProducts, catalog #BBH-75-K]). The protein amount was determined by a Pierce 660 nm Protein Assay Reagent (Thermo Fischer Scientific, catalog #22660). Proteins (10 mg per tissue preparation) were reduced in 1.0 mM dithiothreitol (DTT; Thermo Fisher Scientific, catalog #20290) and alkylated in 5.5 mM chloroacetamide (Sigma-Aldrich, catalog #C0267). Proteolysis was performed with Pierce Trypsin Protease (Thermo Scientific, MS Grade, catalog #90058), and the reaction was stopped with 10% trifluoroacetic acid for HPLC (Sigma-Aldrich, catalog #302031). The peptides were desalted using Sep-Pak C18 Classic Cartridge (Waters, catalog #WAT051910) following the manufacturer's instructions. Using a Concentrator Plus Complete System (Eppendorf AG, catalog #5305000304), the peptide sample was reduced to a final volume of 0.8 mL of affinity precipitation buffer (50 mM Tris-HCl pH 7.4, 10 mM MgCl_2_, Sigma-Aldrich, catalog #63069; 250 μM DTT, 50 mM NaCl). Peptide amount was determined using a NanoDrop2000 spectrophotometer at 280 nm (Thermo Fisher Scientific).

### Mass spectrometry for tissue proteomes

Peptides were analyzed in data-dependent acquisition (DDA) mode using the Exploris 480 fronted with an EASY-Spray Source, coupled to an Easy-nLC1200 HPLC pump (Thermo Fisher Scientific). Peptides were subjected to a dual column setup: an Acclaim PepMap 100 C18 HPLC Column, 75 μm × 20 mm (Thermo Fisher Scientific, catalog #164946); and an EASY-Spray HPLC Column, 75 μm × 250 mm (Thermo Fisher Scientific, catalog #ES902). The analytical gradient was run at 300 nL/min from 5% to 21% Solvent B (95% acetonitrile/0.1% formic acid) for 50 minutes, followed by 10 minutes of 21% to 30% Solvent B, and another 10 minutes of a sawtooth wash (alternating between 5% and 95% Solvent B) to clean the column. Solvent A was water/0.1% formic acid. The instrument was set to 120 K resolution, and the top N precursor ions in a 3-second cycle time were subjected to MS/MS (HCD collision energies (%): 24, 26, 28). Dynamic exclusion was enabled (60 seconds), the isolation width was 1.2 m/z, the MS1 resolution was 120 K, and the MS2 resolution was 60 K (maximum injection time set to “auto”). Aorta and liver peptides were analyzed using a segmented survey scan, that is, 3 separate survey scan injections: m/z 400 to 1100, m/z 400 to 800, and m/z 700 to 1100.

### MS/MS spectral annotation for the tissue proteomes

The spectral processing steps were analyzed using Proteome Discoverer (version 2.5, Thermo Fisher Scientific). The spectra were queried against the Uniprot mouse database (n = 63,603 entries; downloaded January 2022) using the SEQUEST-HT algorithm. Trypsin (full) was set as the digestion enzyme, allowing up to 2 missed cleavages and a minimum peptide length of 6 amino acids. Oxidation (+15.995 Da) of methionine and acetylation (+42.011 Da) of the N-terminus were set as variable modifications. Carbamidomethylation (+57.021 Da) of cysteine was set as a static modification. Spectral search tolerances were 10 ppm for the precursor mass and 20 mmu for HCD spectra. The peptide false-discovery rate (FDR) was calculated using Percolator (target/decoy method), and spectra were filtered based on a 1.0% FDR. Relative quantification was performed by the Feature Mapper and Precursor Ions Quantifier nodes. The maximum retention time shift for chromatographic alignments was set to 10 minutes, and the mass tolerance was set to 10 ppm. Feature linking and mapping retention time tolerance was 0, and mass tolerance was 0 ppm with a signal-to-noise threshold of 5.

### Enzyme-linked immunosorbent assay

Given the limited amount of available plasma, enzyme-linked immunosorbent assays (ELISAs) were possible for only the 3-month diet groups. We pooled 100 μL plasma from 3 to 4 mice from each chow and HFD group. Mice were randomly assigned to each pool. Six pools of plasma were analyzed for each diet using the following assays performed by Eve Technologies Corp using the Luminex 200 system (Luminex Corp/DiaSorin) with Bio-Plex Manager software (Bio-Rad Laboratories Inc).

#### Cardiovascular disease assay

Seven markers were measured in the samples using the Mouse Cardiovascular Disease Panel 1 7-Plex Discovery Assay Array (MDCVD1) as per the manufacturer's instructions for use (MILLIPLEX Mouse CVD Magnetic Bead Panel 1, catalog #MCVD1MAG-77K, Millipore Sigma). The 7-plex consisted of sICAM-1, MMP-9 (gelatinase B), pecam-1, serpin E1 (total)/PAI-1, sE-selectin, sP-selectin, and thrombomodulin. Assay sensitivities of these markers range from 4 to 351 pg/mL. Individual analyte sensitivity values are available in the MilliporeSigma MILLIPLEX protocol.

#### Mouse 32-plex Discovery Assay

Thirty-two markers were measured in the samples using the Mouse Cytokine/Chemokine 32-Plex Discovery Assay Array (MD32) as per the manufacturer's instructions for use (MILLIPLEX Mouse Cytokine/Chemokine Magnetic Bead Panel, catalog #MCYTOMAG-70K, Millipore Sigma). The 32-plex consisted of eotaxin/CCL11, G-CSF/CSF-3, GM-CSF, GROα/CXCL1/KC/CINC-1, GROβ/CXCL2/MIP-2/CINC-3, IFN-γ, IL-1α, IL-1β, IL-2, IL-3, IL-4, IL-5, IL-6, IL-7, IL-9, IL-10, IL-12(p40), IL-12(p70), IL-13, IL-15, IL-17, IP-10/CXCL10, LIF, LIX, MCP-1/CCL2, M-CSF, MIG/CXCL9, MIP-1α/CCL3, MIP-1β/CCL4, RANTES/CCL5, TNF-α, and VEGF-A. Assay sensitivities of these markers range from 0.3 to 30.6 pg/mL. Individual analyte sensitivity values are available in the Millipore Sigma MILLIPLEX protocol.

#### MMP-5-plex Discovery Assay

Five markers were measured in the samples using the Mouse MMP 5-plex Discovery Assay Array as per the manufacturer's instructions for use (MILLIPLEX Mouse MMP Magnetic Bead Panel 3, catalog #MMMP3MAG-79K, MilliporeSigma). The 5-plex consisted of MMP2 (gelatinase A), MMP3 (stromelysin 1), MMP8 (collagenase 2), proMMP9 (gelatinase B), and MMP12 (macrophage metalloelastase). Assay sensitivities of these markers range from 1.6 to 8.4 pg/mL. Individual analyte sensitivity values are available in the Millipore Sigma MILLIPLEX protocol.

### Network analysis

To generate protein-protein interaction networks that link to the Gene Ontology^40^ and Tabula Muris databases,^41^ gene identifiers were entered into the online Enrichr-KG web tool^42^ or the String Database.^43^ Each tool generates graphical representations of pathway enrichment outputs from the available Enrichr libraries.^44^

### Interspecies analysis

To compare our plasma proteomic dataset with previously published human studies, we integrated results from Núñez et al (2022)^45^ and Ganz et al (2016).^46^ Núñez et al applied a MS/MS workflow to quantify circulating proteins associated with subclinical atherosclerosis (plaque score was calculated in carotids, abdominal aorta, and iliofemoral arteries) (n = 444 patients from theProgression of Early Subclinical Atherosclerosis Study and n = 350 patients from the Aragon Worker Health Study, n = 397 patients with subclinical atherosclerosis). Ganz et al analyzed plasma from patients with stable coronary heart disease (CHD) (n = 938 subjects from the Heart and Soul study) or with a history of myocardial infarction, angiographic evidence of at least 50% stenosis in one or more coronary vessels, prior evidence of inducible ischemia by stress testing, or history of coronary revascularization (n = 971 subjects from HUNT3 study). Ganz et al used the SomaLogic/SOMAscan aptamer-based proteomic platform, which measures a predefined panel of affinity-based protein targets, to derive and validate a protein-based cardiovascular risk score.

We compared the proteins identified in our mouse 3-month time point with the human proteins. To do so, for each human dataset, we extracted the protein identifiers and the corresponding genes, and reported effect directions or associations with cardiovascular phenotypes as provided in the original publications. Overall, 854 genes from the Núñez et al^45^ study and 1,090 genes from the Ganz et al^46^ study were included in the comparison. Overlap analyses were performed by intersecting protein lists between 1) the 2 human datasets, and 2) our mouse dataset and each published human dataset. All analyses were conducted using R (version 4.5.0, R Core Team, 2025).

### Statistical analysis

Continuous data are presented using actual data points with mean ± standard error of the mean (SEM). Data were plotted using either GraphPad Prism version 10.4.0 for Windows (GraphPad Software) or R (version 4.5.0) and the RStudio integrated development environment (version 2024.12.0, Posit Team, 2025). For each proteomic analysis, we performed a 2-group comparison (eg, HFD vs chow diet) using the log-transformed protein group means. For each protein, we applied a Student's *t*-test, and the resulting *P* values were adjusted for multiple testing using the Benjamini-Hochberg procedure to control the FDR (reported as *q* values). A *q* value of <0.05 was considered statistically significant (Supplemental Tables 1 and 2 for plasma diet–dependent changes and Supplemental Tables 3 and 4 for plasma time point–dependent changes; Supplemental Tables 5 and 6 for aorta diet–dependent changes at 3 and 6 months respectively^9^; and Supplemental Tables 7 and 8 for liver diet–dependent changes at 3 and 6 months, respectively).^9^ Pearson correlation analysis was used to evaluate the correlation between protein intensities between samples. Correlation coefficients (*r*) were calculated and reported on the plot. Principal component analysis was performed to reduce data dimensionality and identify patterns of variation among samples. For ELISA analysis, proteins with >4 out-of-range (OOR) values across samples were excluded. Values below the limit of detection were imputed using the lower limit of detection (LLOD) for the corresponding protein. For each protein, a Mann-Whitney test was performed (reported as *P* values) using R and the RStudio integrated development environment. A *P* value of <0.05 was considered statistically significant.

### Data availability

The plasma and tissue^9^ mass spectrometry proteomics data have been deposited to the ProteomeXchange Consortium via the PRIDE^47^ partner repository with the dataset identifiers PXD063218 and PXD051108, respectively.

## Results

### The *Ldlr*^*−/−*^ plasma proteome

**Figure 1:**
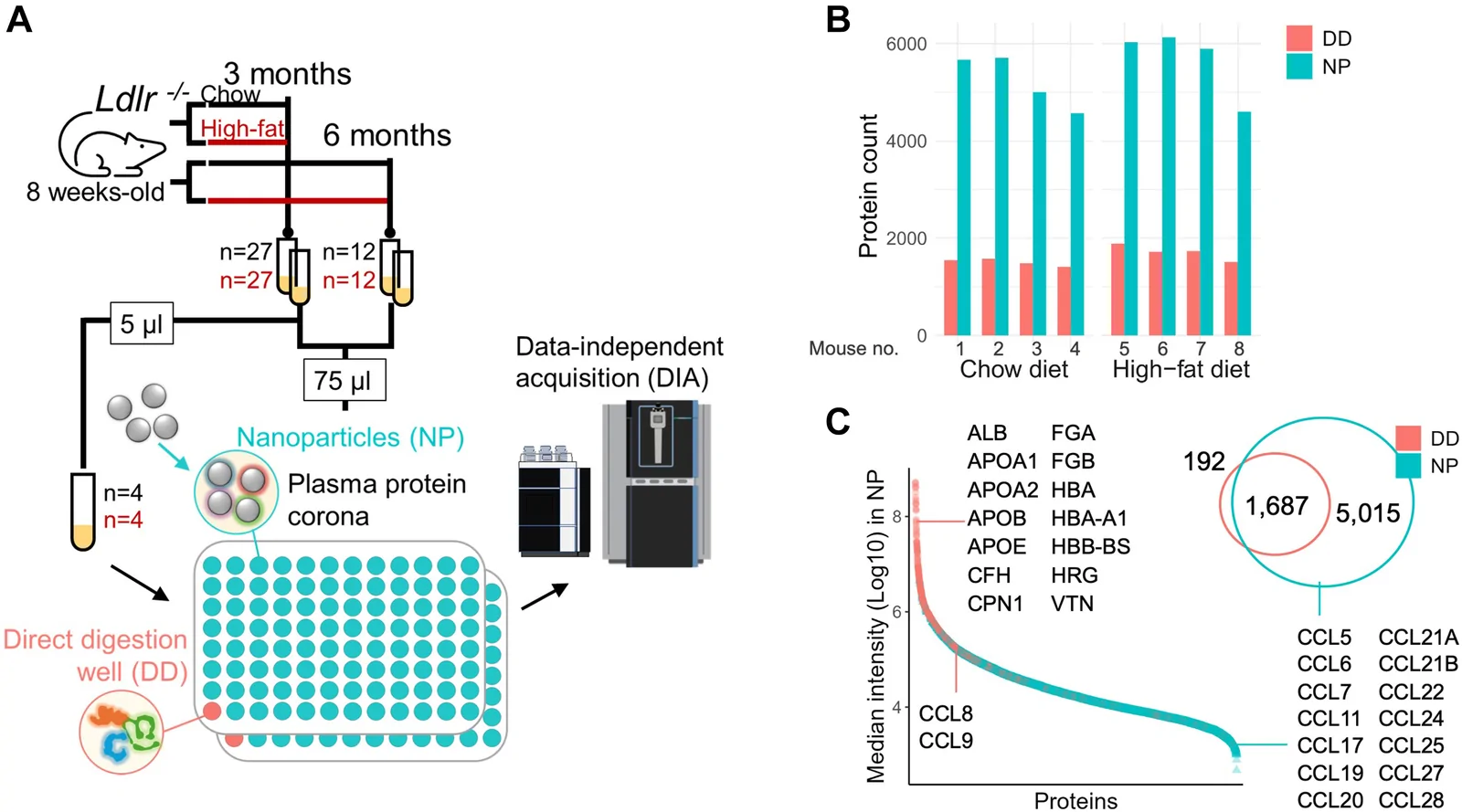
Nanoparticle-Dependent Deep Mouse Plasma Proteomics (Seer's Proteograph Platform) (A) Blood/plasma collection from *Ldlr*^*−/−*^ mice fed a chow or high-fat diet for 3 months (n = 27 mice/plasma samples per diet) or 6 months (n = 12 mice per diet). Eight samples were also processed using a conventional direct digestion protocol. All peptides were analyzed using the Orbitrap Astral in data-independent acquisition (DIA) mode. (B and C) A comparison between the 8 direct digest samples (8 distinct mice plasma) and their corresponding nanoparticle preparations for protein count (B) and median intensity (C). The proteins identified in the direct digestions were mapped to their corresponding ranks in the nanoparticle data. DD = direct digest; NP = nanoparticle.

In this study, blood was drawn from the left ventricle of *Ldlr*^*−/−*^ mice, from which we could allocate 75 μL of plasma for nanoparticle-dependent proteomics. We analyzed plasma at 3 and 6 months of chow diet or HFD feeding, with 27 mice per diet regimen for the 3-month group and 12 mice per diet regimen for the 6-month group. We also aliquoted an additional 5 μL of plasma from 8 mice (3-month group) for a standard proteolysis workflow, referred to as the direct digestion method. The tryptic peptides from each plasma preparation were analyzed on the Orbitrap Astral using data-independent acquisition mass spectrometry (Figure 1A).

We first present the comparisons between the nanoparticle and direct digestion methods prepared from the same 8 *Ldlr*^*−/−*^ mice (Figure 1B). The direct digestion method yielded between 1,409 and 1,733 proteins (including single-peptide hits with an FDR >1%; DIA-NN), whereas the nanoparticles improved proteome depth substantially, achieving between 4,028 to 6,360 proteins across the 8 mice; resulting in an increase ranging from 3- to 3.7-fold relative to the direct digestion method (Figure 1B). The proteins captured from the direct digests map to the most abundant proteins from the nanoparticle method (Figure 1C).

**Figure 2:**
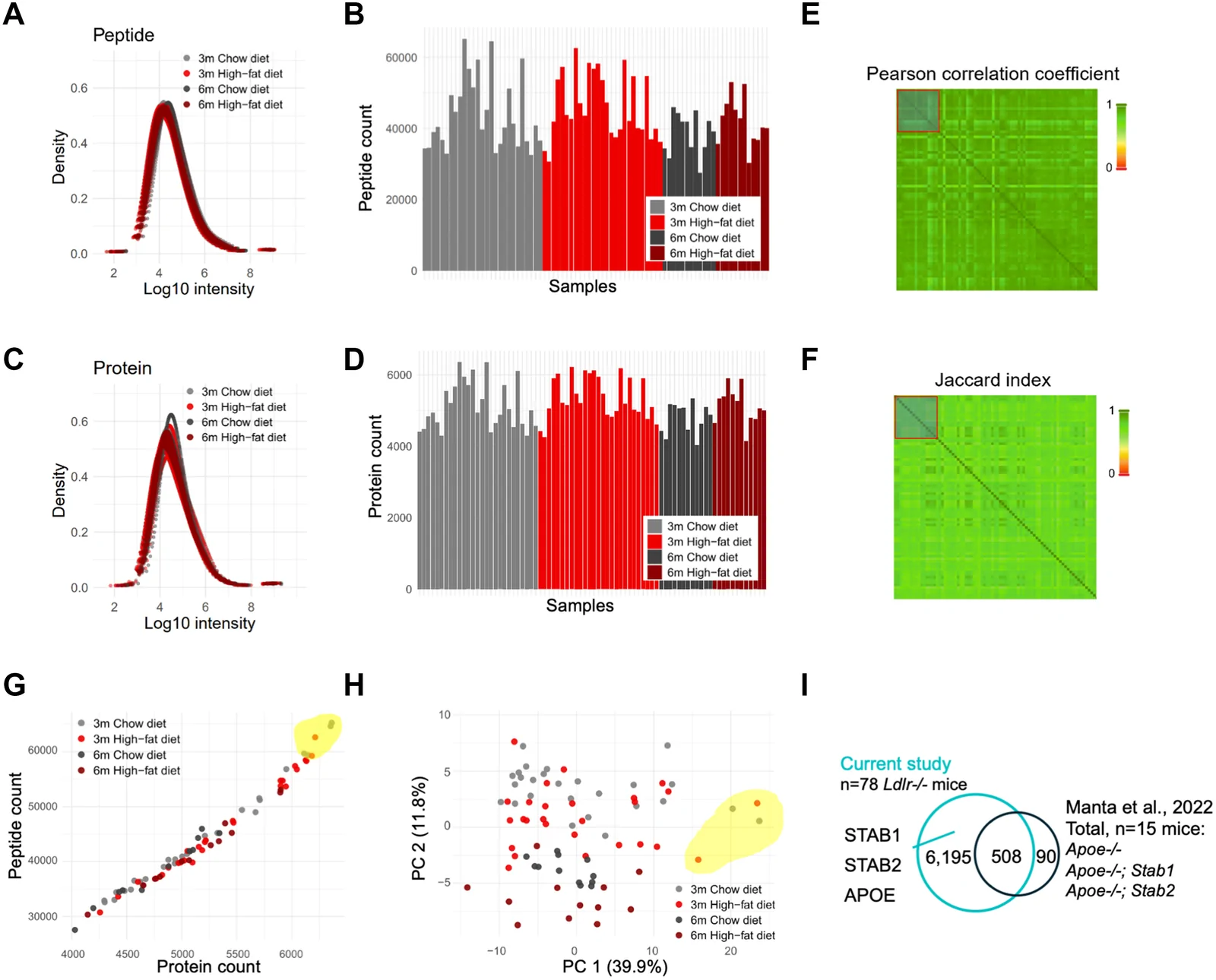
Mouse Plasma Proteome Metrics (A) Distribution of peptide intensities for the 78 mouse plasma samples. (B) The number of peptide identifications across the 78 mouse plasma samples. (C) Distribution of protein intensities for 78 mouse plasma samples. (D) The number of peptide identifications across the 78 mouse plasma samples. (E and F) Sample comparability analysis: statistical correlation of protein intensities between samples based on the Pearson correlation coefficient (E) and the similarity in proteins between samples based on the Jaccard Index (F). (G) Correlation between peptide and protein count (n = 78 samples). (H) Principal component analysis (n = 78) highlighting in yellow that the 4 distinct samples in PC-1 correspond to the samples with the most protein identifications (G)G. (I) Overlap in 2 independent murine plasma proteome studies.

When considering all 78 plasma samples, over 7,000 proteins were identified, and despite being noticeably lipid-laden upon collection and preparation, peptide and protein yields from the HFD plasma samples were comparable to those from chow. For instance, we obtained consistent peptide signal and count for all 78 samples (Figures 2A and 2B), as well as consistent protein signal and count (Figures 2C and 2D). Protein intensity (Pearson correlation coefficient) and similarity (Jaccard index) also revealed comparability between the samples (Figures 2E and 2F). The consistency in these peptide and protein metrics permit us to eliminate sources of technical variation as confounding factors in data analysis.

A few plasma samples yielded an excess of 6,000 proteins, accounting for the most variation in the data (Figure 2H, PC-1); otherwise, the 3- and 6-month groups exhibited visual separation in the principal component analysis (Figure 2H, PC-2). To underscore the advancement in deep mouse plasma proteome profiling, we compared our data with a recent study that employed a magnetic carboxylate-modified bead-enrichment strategy to profile the consensus plasma proteome of three genotypes, including *Apoe*^*−/−*^.^48^ Despite differences in mouse strains, experimental design, and workflows, 85% (508 out of 598) of the proteins identified by Manta and colleagues^48^ were also detected in our data. Unique to our dataset, however, are STAB1 and STAB2, the proteins of interest that were not captured in the previous plasma proteomes (Figure 2I).

### Diet and time–dependent changes to the *Ldlr*^*−/−*^ plasma proteome

**Figure 3:**
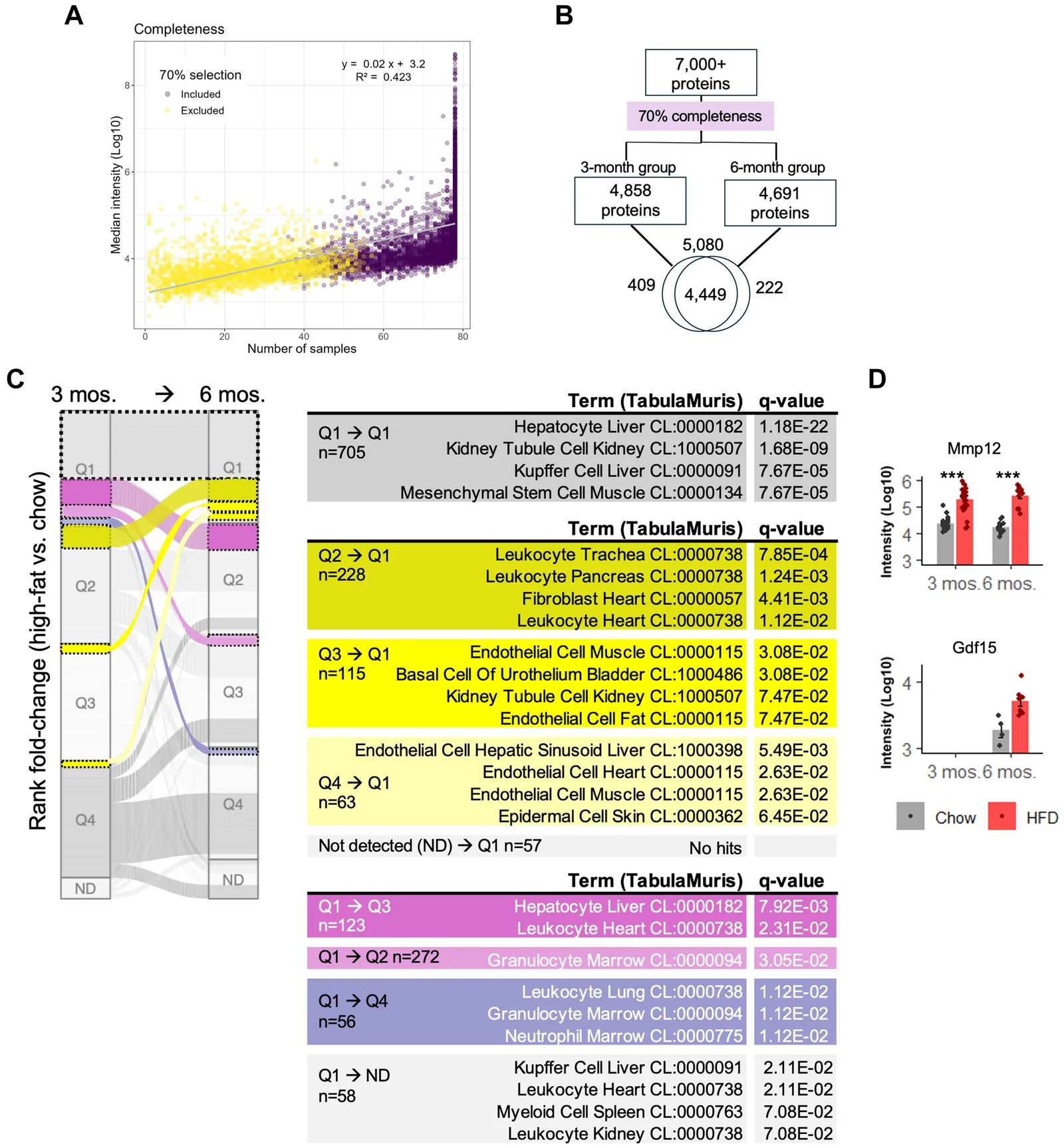
Diet and Time–Dependent Changes to the *Ldlr*^−/−^ Plasma Proteome (A) Protein (median intensities) plotted by the number of samples in which they were identified. Proteins included in 70% of samples (per 3-month and 6-month diet comparison) were likelier to have high-intensity signals. (B) Summary of the final protein sets analyzed in this study, filtered for 70% completeness in each 3-month and 6-month group. (C) Alluvial plot depicting the 3- to 6-month interquartile relationships of proteins arranged by fold-change (high-fat diet vs chow diet). The Tabula Muris single-cell database was queried using proteins from the featured alluvia, and the top 4 cell/tissues are shown (networks with *q* < 0.1 are included). ND = not detected. (D) Bar plots featuring proteins going from Q1 to Q1 (MMP12) and ND to Q1 (Gdf15; *q* = 0.088). Data are presented as individual values with mean ± SEM; ∗∗∗*q* < 0.001. Gray denotes chow diet; red denotes high-fat diet (HFD).

We next filtered the data to exclude proteins that represent a minority of mice (Figure 3A) by applying a 70% completeness threshold^49^ for either diet group per time point comparison; that is, including proteins identified in 19 of 27 for the 3-month diet comparison and 8 of 12 for the 6-month comparison. The filtered proteome was reduced to 5,080 proteins with 4,858 and 4,691 proteins for the 3- and 6-month groups, respectively (Figure 3B).

We first overviewed sweeping HFD and time point–dependent changes to the *Ldlr*^*−/−*^ plasma proteome. We computed the rank of each protein based on its fold-change in response to HFD (relative to chow diet) at 3 and 6 months. Proteins were then aggregated according to their fold-change quartile. To visualize transitions between quartiles over time, we used an alluvial plot (Figure 3C). We focused on proteins whose ranks remained within the top 25th percentile at both time points (Q1), as well as those that migrated from the remaining quartiles at 3-month to Q1 at 6-month (Figure 3C). We extracted the corresponding proteins and queried them into the Tabula Muris single-cell atlas database,^41^ to gain insight into potential source organs or cells. Proteins with a sustained elevation in HFD compared to chow (704 proteins from 3-month Q1 to 6-month Q1) (Figure 3C), associated with markers of liver (hepatocyte and Kupffer cells) but also kidney, mesenchymal stem cells, and fat granulocytes (Figure 3C). The prevalence of liver cells in the top-ranking quartile for both time points is consistent with the liver as a primary source of circulating molecules. The remaining interquartile migrations indicate other source cells and organs. The Q2 to Q1 migration (228 proteins) associates with leukocytes, fibroblasts, and stromal cells, and the Q3 to Q1 (115 proteins) and Q4 to Q1 (63) migrations associate primarily with endothelial cells and monocytes (Figure 3C). These data demonstrate that with high-fat feeding, proteins traditionally associated with endothelial biology become increasingly prevalent in circulation. Of note, proteins that were not detected (ND) at 3 months but appeared in Q1 at 6 months (57 proteins) did not yield any hits, indicating disparate or uncharacterized associations for Tabula Muris. Nonetheless, we noted that growth/differentiation factor 15 (GDF15), a stress response cytokine,^50^ was present in the 3-month ND to 6-month Q1 migration group. GDF15 and macrophage metalloelastase-12 (MMP12; Q1 to Q1) (Figure 3D) are 2 of 8 proteins that were recently shown to associate with incident CHD in 2 distinct prospective study cohorts (the Cardiovascular Health Study and the Atherosclerosis Risk in Communities Study).^51^ Two more of the 8 CHD-associated proteins were also detected in our data, liver-expressed antimicrobial peptide 2 (LEAP2; Q4 to Q1 migration) and DnaJ homolog subfamily B member 9 (DNJB9; Q1 to Q1), however their increased levels at 6 months of HFD were subtle (*q* = 0.163 and *q* = 0.220, respectively). We also profiled the proteins that migrated from Q1 at 3 months to one of the lower quartiles including the not detected group (Q1 to ND) at 6 months. These proteins primarily associated with leukocytes (Figure 3C), specifically enriching the granulocytes/neutrophils subtypes, suggesting the contribution of neutrophils and other granulocytes are diminished in later stages of disease.^52^^,^^53^

### Peroxisomal and mitochondrial-associated proteins are enriched in HFD *Ldlr*^*−/−*^ plasma

**Figure 4:**
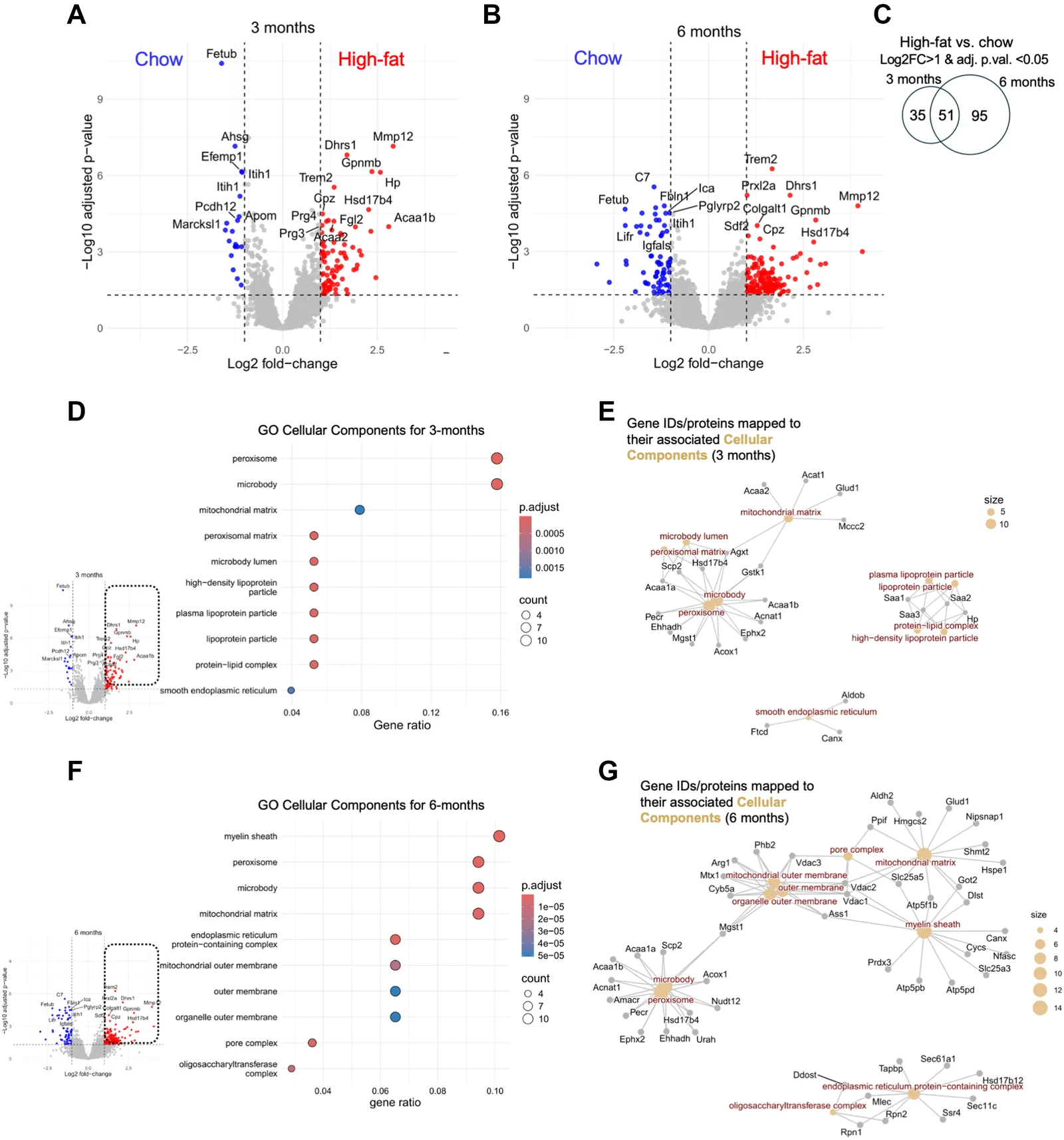
High Fat Diet–Induced Changes to the *Ldlr*^−/−^ Proteome and Associated Cellular Components (A and B) Volcano plots depicting the group mean comparisons (ie, mean high-fat diet [HFD] vs mean chow diet) for each 3-month and 6-month time point. Red and blue points denote proteins with log2 fold-change >1 (red) or < 1 (blue) (*q* < 0.05); n = 27 mice per 3-month group, and n = 12 mice for each 6-month group. (C) A comparison of the uniquely and commonly identified HFD-associated protein abundances from (A) and (B). (D and F) Gene Ontology (GO) analyses for the HFD-associated increases in the *Ldlr*^*−/−*^ proteome at 3 months (D) and 6 months (F). Miniature volcano plots are from panels (A) and (B). The top 10 cellular components are listed. (E and G) Corresponding networks depicting the protein/gene identifiers (IDs) to cellular component associations for (D) and (F).

We then evaluated the effects of diet by comparing the means of each 3-month (27 mice per diet) and 6-month (12 mice per diet) group (Figures 4A and 4B). At 3 months on an HFD, 86 proteins increased and 20 decreased; at 6 months, 146 proteins increased and 49 decreased. Proteins were considered differentially abundant based on a log_2_ fold-change greater than 1 or lower than −1 (*q* < 0.05). Two proteins that stand out are MMP12, which increased in HFD at both time points, and fetuin-B, a protease inhibitor, which decreased at both time points. Peroxisomal 3-ketoacyl-CoA thiolases A and B (ACAA1a/b) and procollagen galactosyltransferase 1 (COLGALT1) represent proteins unique to each 3- and 6-month filters (Figures 4A and 4B). Of the increased proteins, 51 (28%) are common to both time points, including MMP12, peroxisomal acyl-CoA oxidase (ACOX1), mitochondrially encoded cytochrome c oxidase II (MTCO2), or the mitochondrial matrix enzyme glutamate dehydrogenase 1 (GLUD1). This overlap is encouraging, especially considering that the 3-month and 6-month plasma samples were not paired; the mice came from separate experiments, as each time point involved a terminal sacrifice.

We examined more closely the top-ranking HFD-associated proteins (log_2_ fold-change <1, *q* < 0.05) for each time point using the Gene Ontology (GO) Cellular Components database (Figures 4D to 4G). At 3 months, the top 10 cellular components retrieved from our protein lists associated primarily with mitochondrial and peroxisomal compartments, but also lipoproteins (Figures 4D and 4E). The lipoproteins terms are not surprising, as they comprise the most abundant particle in plasma, but it is interesting to note that it is the serum amyloid proteins (SAA1, SAA2, and SAA3) that are associated with this ontology (Figure 4E). Proinflammatory conditions increase the prevalence of HDL-associated serum amyloids, decreasing the particle's antiatherogenic potential.^54^ The presence and increased abundance of mitochondrial and peroxisomal proteins are also interesting, as these findings demonstrate that their proteins are secreted into circulation. At 6 months, the top 10 cellular components are also associated with peroxisomes and mitochondria, but in this case, additional proteins indicative of membrane fractions are enriched (Figures 4F and 4G). Taken together, the Tabula Muris (Figure 3C) and GO enrichments (Figures 4D and 4G), HFD feeding of *Ldlr*^*−/−*^ mice may promote membranous or vesicle-mediated release of peroxisomal and mitochondrial proteins.

### The apolipoproteins exhibit distinct responses to HFD feeding

**Figure 5:**
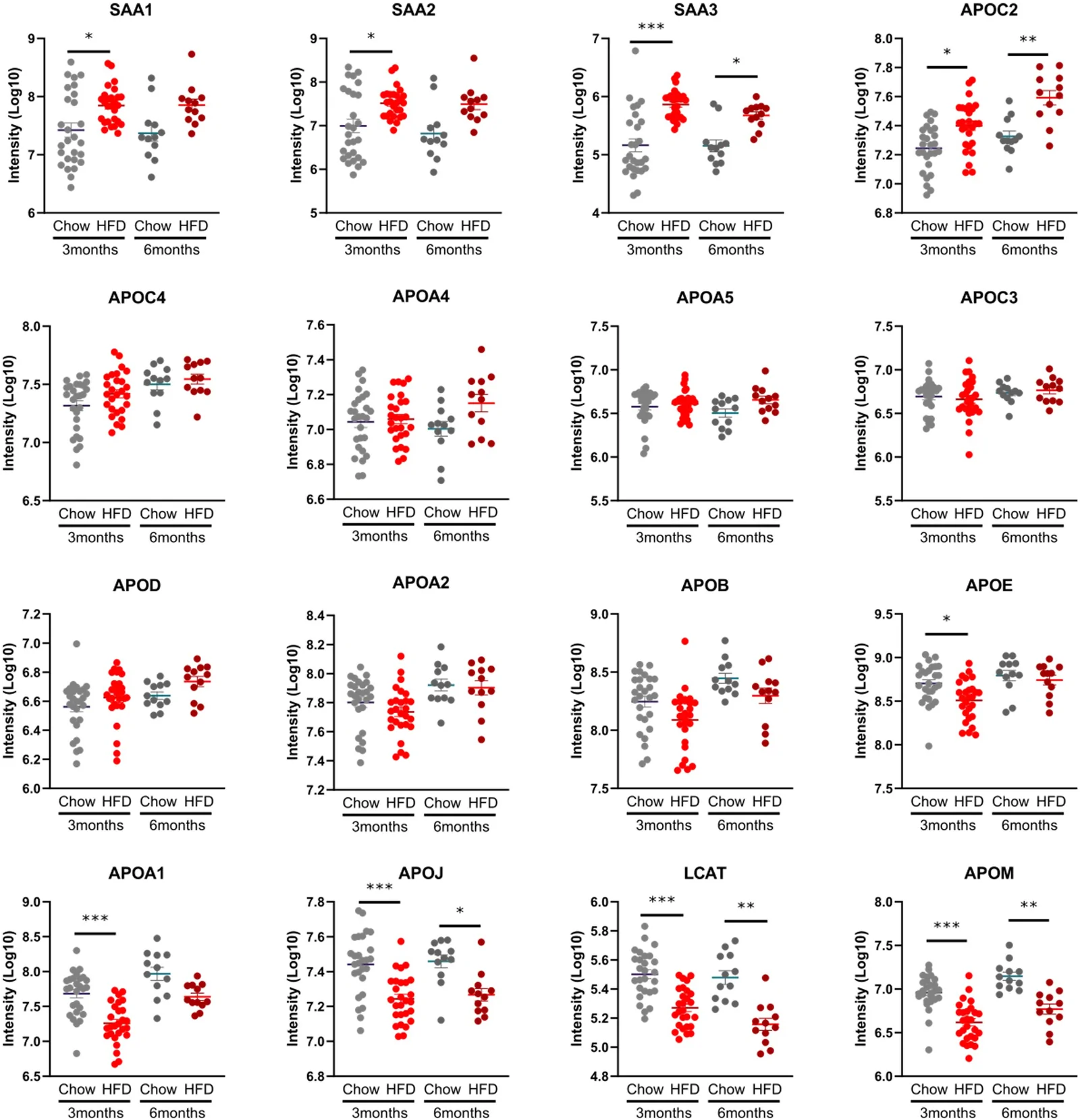
*Ldlr*^*−/−*^ Plasma Apolipoprotein Profiles Featured apolipoproteins are ordered from increasing to decreasing abundances associated with high-fat diet (HFD) feeding. Student's *t*-test for each protein's comparison and the Benjamini-Hochberg procedure to calculate *q* values. Data are presented as mean ± SEM; ∗*q* < 0.05, ∗∗*q* < 0.01, and ∗∗∗*q* < 0.001. Gray denotes chow diet; red denotes high-fat diet.

Approximately 100 proteins form or bind to circulating lipoproteins in mice and humans, including the serum amyloid proteins.^55^^,^^56^ We extracted the abundance profiles of some of the classical apolipoproteins^57^ identified in our data, and arranged them from increasing to decreasing HFD-associated changes (Figure 5). SAA1, SAA2, and SAA3 increased with HFD at 3 and 6 months of feeding, but only SAA3 surpassed the statistical cutoff (*P*_adjusted_ < 0.05) at both time points (Figure 5). Of the remaining apolipoproteins in our analysis, only APOC2 exhibited a similar profile to SAA3 (Figure 5). On the other hand, APOE, APOA1, APOJ, LCAT (phosphatidylcholine-sterol acyltransferase), and APOM decreased with HFD, at one or both time points (Figure 5). APOA2, APOA4, APOA5, APOC3, APOC4, APOD, and APOB remained constant between chow and HFD (Figure 5). APOA1 is the main structural protein for HDL, and LCAT is a hallmark lipid metabolizing enzyme residing on HDL.^58^ Thus, the decrease in APOA1 and LCAT, combined with the increases in the serum amyloids together, may indicate a loss of normal HDL function with high-fat feeding.^59^, ^60^, ^61^

### Plasma reflects tissue proteins linked to immunity, ECM components, and lipid metabolism

We compared *Ldlr*^*−/−*^ plasma proteome to those defining the diseased liver and aorta (ie, fatty liver and atherosclerotic aorta). The liver and aorta samples were collected using conventional (without nanoparticle technology) proteomics^9^^,^^36^^,^^39^; therefore, differences in the sample processing and protein yields exist. Ten aortas were pooled for each biological replicate (n = 4 replicates) per diet. Meanwhile, 1 liver sample was acquired from 6 independent mice per diet. The aorta proteome (≥2 unique peptides; chow and HFD combined) comprises 1,548 proteins, and the liver proteome, 2,142 proteins. While 33% of the plasma proteome (1,622/4,858 total plasma proteins) was shared with liver and/or aorta, plasma shared more proteins with the liver than the aorta (Supplemental Figure 1A). We used the GO analysis via the String Database to summarize the top 5 biological processes associated with these overlapping proteins (Supplemental Figures 1B to 1E). For proteins common to plasma and aorta, most of them are associated with ECM functions, namely the collagens and MMPs (Supplemental Figures 1B and 1C).

**Figure 6:**
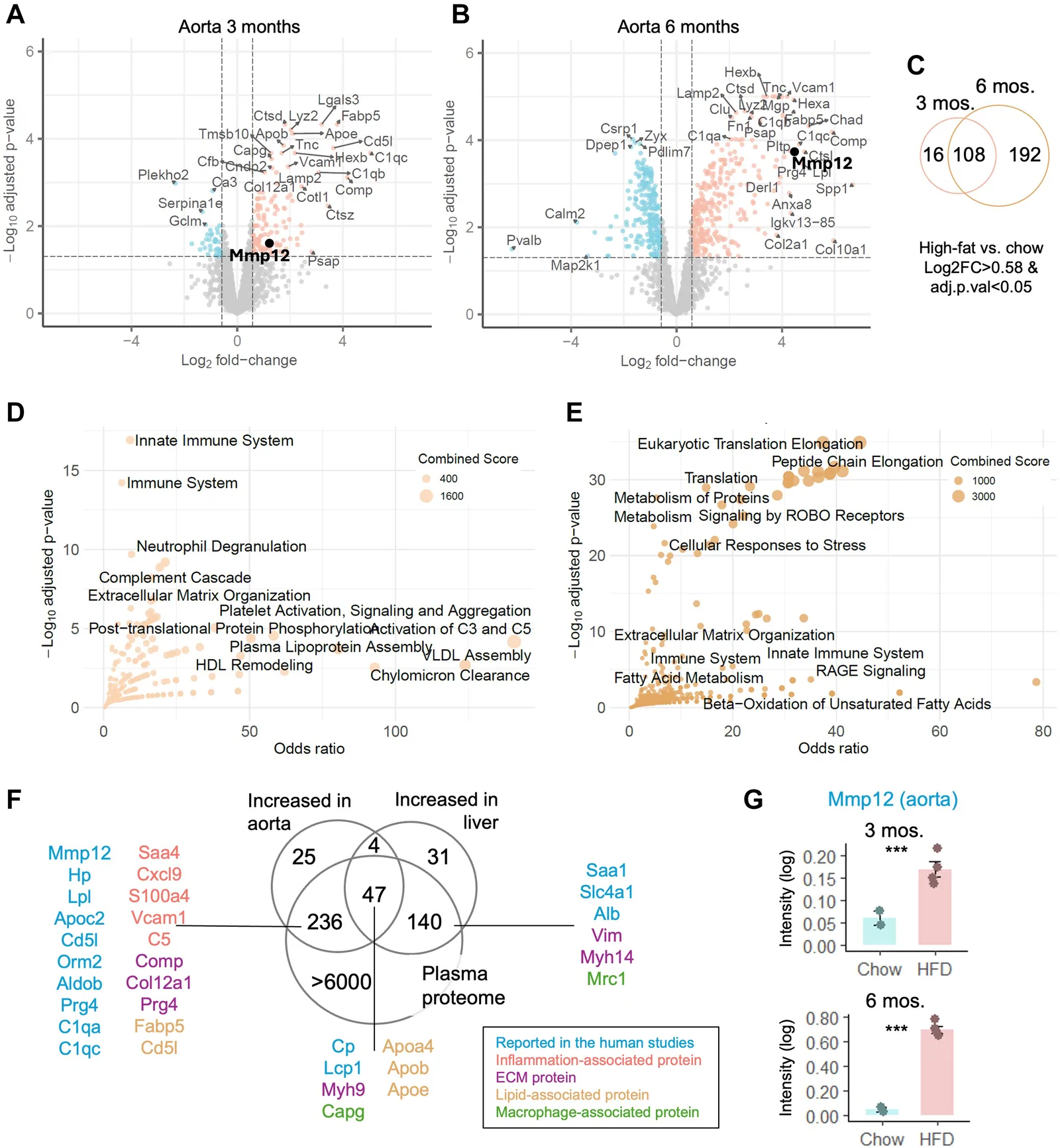
High-Fat Diet–Induced Changes to the *Ldlr*^−/−^ Aorta Proteome and Associated Pathways (A and B) Volcano plots depicting the group mean comparisons (ie, mean high-fat diet [HFD] vs mean chow diet) for each 3-month and 6-month time point. Orange and blue points denote proteins with log2 fold-change >0.58 (orange) or <−0.58 (blue) (*q* < 0.05); n = 4 aorta pools per group. (C) A comparison of the uniquely and commonly identified HFD-associated protein abundances from (A) and (B). (D and E) Reactome enrichment analysis for the HFD-associated increases in the *Ldlr*^*−/−*^ proteome at both 3 and 6 months (D) and exclusively at 6 months (E). (F) A comparison of the uniquely and commonly identified HFD-associated protein in aorta (312 proteins) and liver (222 proteins), with all protein identified in plasma. Both time points are combined for each tissue. (G) Bar plots featuring MMP12 abundance in the aorta. Data are presented as individual values with mean ± SEM; ∗∗∗*q* < 0.001. Blue denotes chow diet; red denotes high-fat diet.

We evaluated the effects of diet on the aorta by comparing the means of each 3- and 6-month group (Figures 6A and 6B). At 3 months of HFD, 124 proteins increased and 38 decreased; at 6 months, 300 proteins increased and 235 decreased. Proteins were considered differentially abundant based on a log_2_ fold-change greater than 0.58 or lower than −0.58 (*q* < 0.05). HFD induced an increase in several known mediators of plaque progression including MMPs (MMP12, MMP2), hemoglobin-binding haptoglobin protein (HP),^62^^,^^63^ cell adhesion proteins (VCAM1),^64^ inflammatory proteins (CRP),^65^ and apolipoproteins (APOB).^66^ Most of the proteins that increased at 3 months remained elevated at 6 months (108 of 124 proteins at 3 months also increased at 6 months) (Figure 6C). We then queried the Reactome database (via EnrichR, Methods) for proteins enriched in HFD vs chow diet, revealing an over-representation of immune response pathways, ECM organization, platelet activation, and lipid metabolism at both time points; but with pathways associated with translation uniquely defining the 6-month HFD-increased proteins (Figures 6D and 6E). We conducted a similar interdiet comparison with the liver proteomes (Supplemental Figures 2A and 2B), noting that proteins associated with lipoprotein assembly and metabolism of lipids increased with HFD at both time points; whereas proteins associated with innate immunity are uniquely prominent at 6 months (Supplemental Figures 2C and 2D). When considering the 3- and 6-month HFD-induced proteins for each aorta (312 proteins) and liver proteome (222 proteins), 236 aorta and 140 liver proteins were identified in plasma and 47 were identified in all 3 tissues (Figure 6F). Some of these shared proteins are highlighted, including those that have been reported in the human cardiovascular plasma studies that we detail further below, such as MMP12,^51^ HP, and lipoprotein lipase (LPL) for the aorta-plasma overlap, and SAA1 for the liver-plasma overlap (Figure 6F). Of particular interest is MMP12, which is markedly increased in the aorta (Figure 6E). We also processed a subset of the 3-month plasma samples to measure with ELISA not only MMP12 but other cytokines, most of which were not detected in the proteomics, for example, monocyte chemoattractant protein-1 (MCP-1) and the interleukins (IL-4, IL-5) (Supplemental Figure 3). Seven assayed proteins significantly increased with HFD: MMP12 and MCP-1 (*P* < 0.01), and vascular endothelial growth factor (VEGF), IL-4, IL-13, plasminogen activator inhibitor 1 (PAI-1/SERPINE1) (*P* < 0.05), and monokine induced by gamma interferon (MIG/CXCL9) (*P* < 0.05). Three of these ELISA proteins were also detected in the proteomics, and all increased at both time points with HFD: MMP12 (*q* < 0.001) and PAI-1 (*q* < 0.001), or at 3 months: CXCL9 (*q* = 0.187).

### Genes/proteins implicated in coronary artery disease are detected in *Ldlr*^*−/−*^ plasma

**Figure 7:**
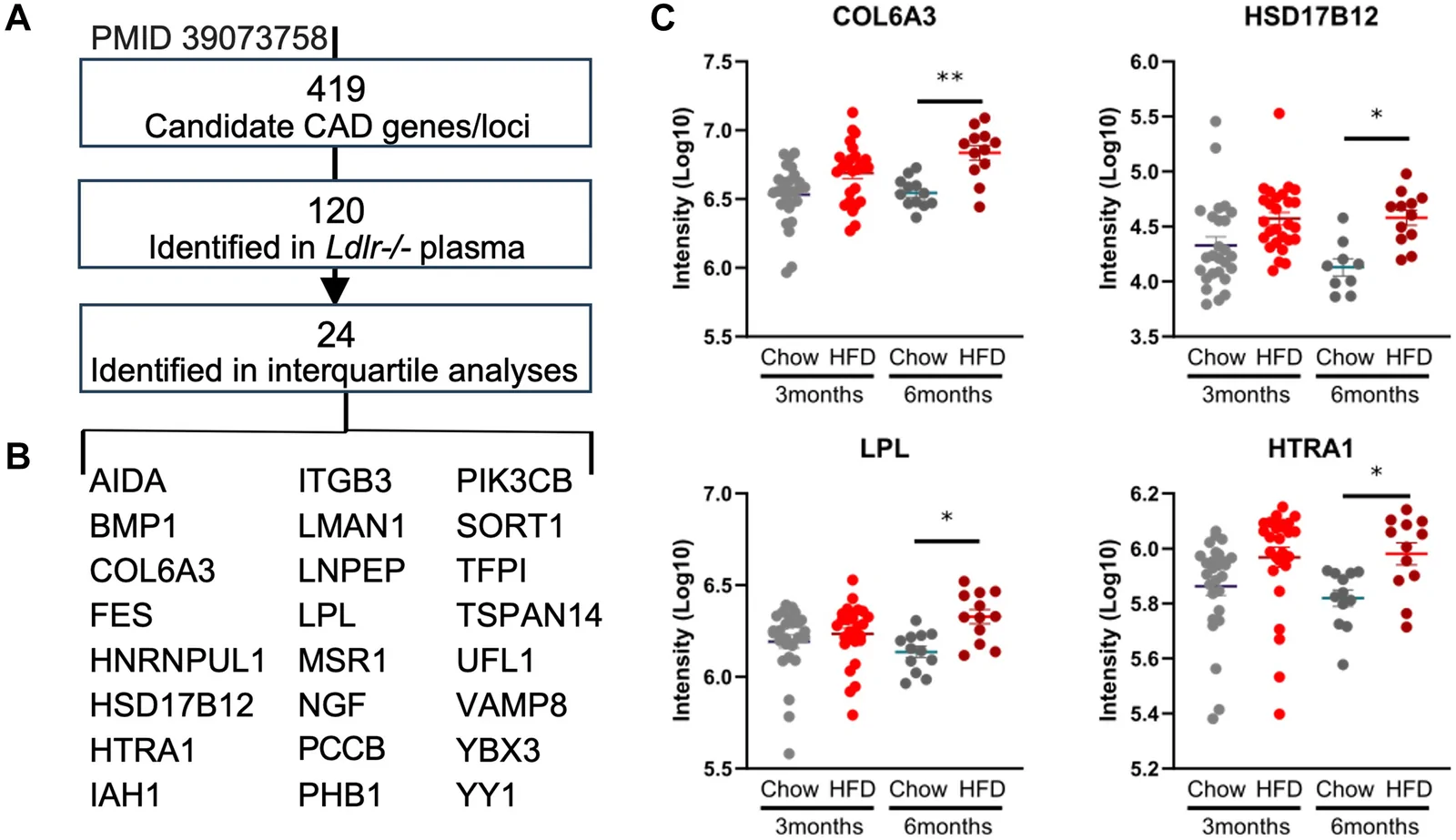
Candidate Coronary Artery Disease–Associated Genes/Proteins Identified in *Ldlr*^*−/−*^ Plasma (A) List of candidate CAD-associated loci/genes curated by Aherrahrou et al^67^ (2024), cross-referenced with the proteins identified in our study. (B) A total of 24 proteins were extracted from the interquartile analysis in Figure 2. (C) Proteins that increased in high-fat diet over chow at one or both time points. Student's *t*-test for each protein's comparison and the Benjamini-Hochberg procedure to calculate the *q* value. Data are presented as mean ± SEM; ∗*P* < 0.05, ∗∗*P* < 0.01, and ∗∗∗*P* < 0.001. CAD = coronary artery disease; HFD = high-fat diet.

Although disease progression in *Ldlr*^*−/−*^ mice does not fully reflect the human condition,^8^ preclinical models are nonetheless essential for developing and testing candidate therapies. Human genome-wide association studies have implicated several candidate genes in coronary artery disease (CAD).^67^ We accessed a list of these CAD-associated genes curated by Aherrahrou et al,^67^ of which most were supported by eQTL data, and confirmed that 120 of the 419 genetic products were detected in the *Ldlr*^*−/−*^ plasma proteome (Figure 7A). Of these 120 proteins, 24 were detected in our interquartile range analysis (Figure 3C) and 4 exhibited marked increase in abundance with HFD: LPL, very-long-chain 3-oxoacyl-CoA reductase (HSD17B12), serine protease HTRA1 and collagen alpha-3(VI) chain (COL6A3) increased significantly at 6 months (*P*_adjusted_ < 0.05) (Figures 7B and 7C). Whether the plasma profiles of these 4 or the remaining 116 proteins provide insight or meaning into the human condition is currently unknown. However, the ability to detect them in *Ldlr*^*−/−*^ mouse plasma positions us to monitor them in future preclinical studies.

### Cross-species comparison reveals common plasma proteomic signatures between *Ldlr*^−/−^ mice and human vascular disease

**Figure 8:**
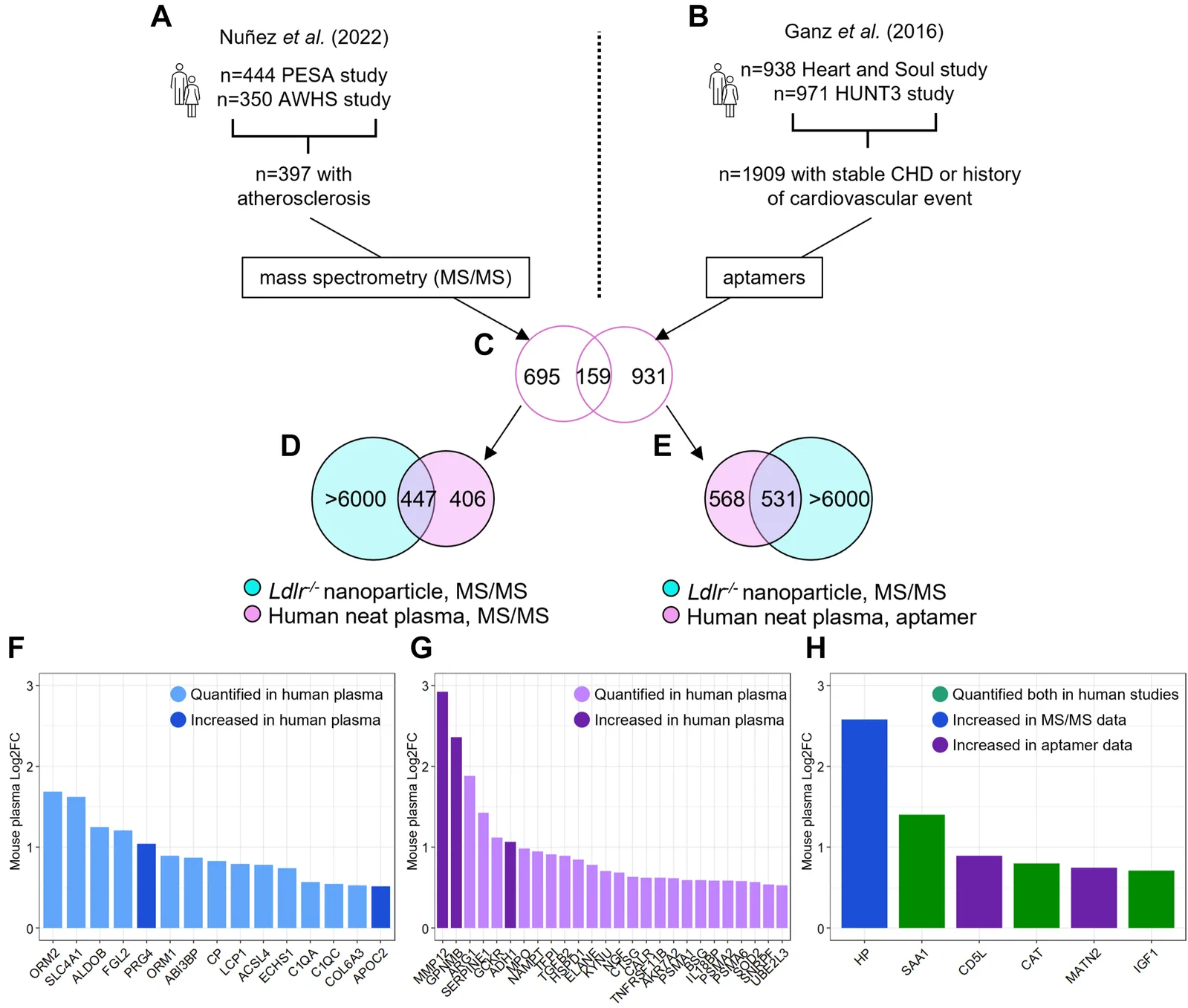
Cross-Species Comparison of Mouse and Human Plasma Proteomes in Atherosclerosis and Coronary Heart Disease Context (A to C) Integration of the mouse plasma proteome with existing human plasma proteomic datasets from individuals with subclinical atherosclerosis (Núñez et al,^45^ 2022) or coronary heart disease (CHD) (Ganz et al,^46^ 2016). (A) Overview of the human cohort and proteomic approach used in Núñez et al. (B) Overview of the human cohort and proteomic method used in Ganz et al. (C) Comparison of the plasma proteins quantified in the 2 human studies. (D) Venn diagram showing the overlap between proteins identified in the mouse plasma (this study) and those detected in the human subclinical atherosclerosis cohort. (E) Venn diagram showing the overlap between proteins identified in the mouse plasma (this study) and those detected in the human CHD study. Integration of the mouse plasma proteome with an independent human proteomic dataset from patients with CHD quantified using SomaLogic/SOMAscan aptamer-based technology. (F to H) Mouse log2FC of proteins identified in humans and increased in the mouse (log2FC >0.5 and *P*_adjusted_ < 0.1) in the MS/MS subclinical atherosclerosis study (F), the SomaLogic CHD (G), and common to both studies (H). AWHS = Aragon Worker Health Study; MS/M = Standem mass spectrometry; PESA = Progression of Early Subclinical Atherosclerosis Study.

To further evaluate the translational relevance of the *Ldlr*^*−/−*^ mouse plasma proteome, we compared our data to 2 independent human proteomic datasets generated in the context of vascular disease.^45^^,^^46^ The first study (Núñez et al^45^) profiled plasma from individuals with subclinical atherosclerosis (in carotids, abdominal aorta and iliofemoral arteries) using a mass spectrometry approach that did not entail nanoparticle-dependent proteome enrichment (Figure 8A); whereas the second study (Ganz et al^46^) quantified proteins in patients with CHD using the SomaLogic/SOMAscan aptamer platform (Figure 8B). The mass spectrometry proteome comprised 854 proteins representing 367 patients, and the aptamer panel targeted 1,090 plasma proteins representing 1,909 patients. The overlap between the 2 datasets is limited (159 shared proteins) (Figure 8C), which is more likely because the 2 technologies analyzed distinct dynamic ranges within the plasma proteome, rather than due to the distinct patient cohorts.

We next examined the extent to which proteins identified in our mouse dataset were also detected in either of the human studies. A subset of proteins (447 proteins) was shared between mouse plasma and the mass spectrometry–based subclinical atherosclerosis cohort (Figure 8D), while another subset (531 proteins) overlapped with the CHD dataset quantified by the aptamer technology (Figure 8E). The nanoparticle-enriched workflow yielded an unprecedented depth of the mouse plasma proteome, identifying 6 times more proteins than either human dataset. Despite the differences in disease, disease stage, analytical platform, and yield, proteins that increased in our mouse model (log_2_FC >0.5, *P*_adjusted_ < 0.1) were likewise identified or increased in the human studies. These included proteins uniquely reported in the mass spectrometry subclinical atherosclerosis dataset (lipid-related proteins APOC2, ACSL4; complement components C1QA, C1QC),^68^^,^^69^ (Figure 8F), those specifically detected in the CHD aptamer panel (proteasome complex proteins PSMA1, PSMA2, PSMA6; interleukin family protein IL18BP; matrix-related proteins CTSG, MMP12; TGFB2; SERPINE1)^37^; (Figure 8G), and a group common to both human cohorts (acute-phase proteins HP, SAA1) (Figure 8H). Pathological roles in cardiovascular disease of some of these proteins are already reported, highlighting the clinical relevance of the mouse plasma data. Together, these cross-species comparisons highlight the emergence of conserved plasma signature linking inflammation-associated proteins in our mouse model to human atherosclerosis and CHD.

## Discussion

This study presents the most comprehensive plasma proteome of experimental atherosclerosis, specifically the *Ldlr*^−/−^ mouse model, a widely used preclinical species for studying the pathogenesis of atherosclerosis and metabolic disorders. High-fat feeding for 3 to 6 months in 8- to 10-week-old *Ldlr*^−/−^ mice was sufficient to induce aortic lesions, as previously demonstrated,^9^^,^^14^^,^^19^^,^^30^^,^^31^ increasing the chance to confirm the molecular signatures that we also profiled in the diseased liver and aorta. Our study design highlights the benefits of including these diseased tissues to assess the relevance of circulatory profiles; but also, it demonstrates that future studies aiming to profile earlier stages of disease (eg, up to 3 months of HFD feeding) in which molecular features may be distinct from later stages and less prominent, should enlist a multiorgan study design with more than 10 animals per diet group.

To conduct deep plasma proteomics, we relied on recently developed nanoparticle-dependent protein corona-forming technologies that have only been reported for mostly human studies.^24^ We quantified over 5,000 proteins representing 78 *Ldlr*^−/−^ mice analyzed at either 3 or 6 months of chow or high-fat feeding, surpassing a previous *Apoe*^−/−^ plasma report by 10-fold.^48^ In addition, the recent advent of the Astral mass analyzer that, when implemented as a hybrid mass spectrometer, provides both fast and sensitive performance scans optimal for unprecedented comprehensive proteomics.^70^^,^^71^ Only 75 μL of plasma (used for 2 assays per mouse) were sufficient for profiling. We isolated plasma from terminal blood draws of 1,000 to 2,000 μL; however, nonterminal studies that rely on tail vein or retrobulbar venous sinus bleeding can draw up to 250 μL of blood, which is sufficient for our workflow.

The marked increase in MMP12 at both 3 and 6 months of high-fat feeding is particularly striking, and it is noteworthy, as it is also elevated in the aorta. Plasma MMP12 levels are higher in individuals with progressive atherosclerosis, aortic valve disease, and abdominal aortic aneurysm,^72^, ^73^, ^74^, ^75^, ^76^, ^77^ implicating MMP12 as a potential biomarker for vessel disease progression in at least the *Ldlr*^−/−^ mice. An aptamer-based plasma proteomic analysis of subjects enrolled in 2 prospective study cohorts, the Cardiovascular Health Study and the Atherosclerosis Risk in Communities Study, demonstrated that plasma MMP12 exhibited the strongest association with incident CHD.^51^ Interestingly, this association was attenuated with subclinical atherosclerosis, that is, measures of ankle-brachial index, carotid artery stenosis, and carotid intima media thickness; and multiple genome-wide association study analyses indicated that genetically predicted, higher MMP12 is protective for CHD.^51^ This prospective study analysis underscores that, even for the proteins identified in our study, associations with disease progression alone are insufficient to establish causality. Further mechanistic studies could elucidate whether MMP12 directly contributes to plaque instability or merely reflects inflammatory remodeling, thus refining its utility as a therapeutic target or biomarker.

Apolipoproteins revealed distinct patterns of response to HFD feeding in *Ldlr*^−/−^ mice, reflecting the complex alterations in lipoprotein metabolism during atherogenesis. The observed decrease in APOA1 and LCAT is notable given their pivotal roles in HDL particle formation and cholesterol efflux. APOA1 is the major structural protein of HDL, and LCAT is critical for HDL maturation and reverse cholesterol transport; reductions in these proteins likely impair HDL's atheroprotective functions, consistent with prior reports that HDL dysfunction accompanies CAD.^78^, ^79^, ^80^ Conversely, the increase in plasma serum amyloid proteins SAA1-3 further suggests a shift towards a proinflammatory HDL phenotype, known to reduce HDL's anti-inflammatory and antioxidant properties.^81^ Of note, APOC2, a cofactor for LPL, also increased with HFD, potentially indicating an adaptive response to elevated triglyceride-rich lipoproteins. These dynamic changes underscore the importance of apolipoprotein remodeling in the pathogenesis of diet-induced atherosclerosis.

The hepatokine fetuin-B, a member of the superfamily of cysteine protease inhibitors, decreased in plasma with HFD. Fetuin-B is involved in metabolic regulation and inhibition of ectopic calcification, but its role in the cardiovascular context remains less clear that that of fetuin-A.^82^ Decreased circulating fetuin-B levels could reflect hepatic dysfunction or altered systemic inflammatory states associated with diet-induced atherosclerosis. Recent studies suggest fetuin-B may influence insulin resistance and lipid metabolism,^83^ linking it to metabolic pathways that intersect with atherogenesis.^84^ Our data position fetuin-B as a novel candidate for further exploration and a possible modulator of metabolic and inflammatory aspects of cardiovascular disease. These findings advocate for the utility of combining plasma proteomics with tissue profiling to improve biomarker discovery and to elucidate the crosstalk between metabolic and vascular compartments in atherosclerosis.

Our study provides, to our knowledge, the first systematic cross-species comparisons of circulating plasma proteomic signatures between experimental atherosclerosis in mice and human vascular disease cohorts. Despite differences in species, disease stage, and analytical platforms, approximately half of the proteins detected in either human dataset were also identified in the *Ldlr*^−/−^ mouse plasma proteome. Shared proteins included factors with established roles in cardiovascular disease, such as mediators of extracellular matrix remodeling and fibrosis (MMP12, TGFB2, PAI-1),^37^^,^^85^ inflammation and innate immunity (HP, CP),^86^ lipid metabolism (APOC2),^87^ and oxidative stress (MPO, SOD2).^88^^,^^89^ In addition, several proteins with emerging or less well-characterized roles in cardiovascular pathology, including FGL2 and SOD2, were common to both species, suggesting that the mouse plasma proteome captures both known and potentially novel disease-relevant signals.

Notably, the limited overlap between the 2 human proteomic datasets themselves highlights the strong influence of analytical technology on plasma protein detection. Mass spectrometry–based approaches and affinity-based platforms interrogate distinct subproteomes and dynamic ranges, which likely accounts for much of the discrepancy observed between human studies. In this context, the convergence of mouse plasma proteins with signals detected across different human cohorts and platforms strengthens the biological relevance of the shared signatures. Nonetheless, these comparisons should be interpreted with caution. Firstly, cross-platform validation remains essential, and mass spectrometry–based approaches are particularly valuable for confirming findings derived from affinity-based technologies.^23^^,^^29^^,^^90^ Secondly, we envision the *Ldlr*^*−/−*^ plasma proteome as a new resource to further characterize this model of atherosclerosis, rather than a direct segue into the human circulatory system. We have yet to understand how the circulatory system reflects the molecular features and mechanisms that drive disease in, in this case, mouse atheroma. Thirdly, we envision that nanoparticle-enabled deep plasma proteomics will eventually be implemented into a comparable clinical setting, providing us with better suited opportunity to compare our findings in experimental atherosclerosis in mice with the human indication.

### Study limitations

Despite substantial technical and biological advances, certain limitations remain outside the scope of this study. The *Ldlr*^−/−^ mice, while highly informative, may not fully capture the complexity of human atherosclerosis. Species-specific differences in proteomic profiles warrant cautious interpretation, as do potential variations introduced by the choice of mouse strains (eg, *Apoe*^*−/−*^ vs *Ldlr*^*−/−*^*).* Nonetheless, our findings provide a valuable resource to support ongoing efforts to bridge preclinical and clinical research, especially biomarker and therapeutic target discovery and validation. Future studies integrating complementary omics approaches, such as lipidomics and transcriptomics, will further strengthen these discoveries and deepen mechanistic insights.

## Conclusions

We demonstrate that nanoparticle-dependent enrichment of low-abundant plasma proteins coupled to mass spectrometry for proteome sequencing offers a viable and powerful strategy to monitor disease-relevant molecular signals in a mouse model of cardiometabolic disorders (eg, dyslipidemia, atherosclerosis, and fatty liver). This study lays the groundwork for future efforts to assess the predictive value of circulating proteins in atherosclerosis and to test whether they can serve as readouts for therapeutic efficacy in preclinical intervention studies. Ultimately, such proteomic strategies could accelerate the translation of preclinical findings to clinical diagnostics and for cardiovascular disease.

## Funding Support and Author Disclosures

This work is supported by grants from the National Institutes of Health (R01HL126901, R01HL149302 to Dr M. Aikawa and R01HL174066 to Drs M. Aikawa, E. Aikawa, and Singh), the American Heart Association (2024A003947 to Dr Singh), Leducq Foundation Transatlantic Network – PRIMA to Dr E. Aikawa, and Kowa Company Ltd (Nagoya, Japan; to Dr M. Aikawa). Kowa played no role other than funding and did not participate in the study design, data collection, analysis, publication decision, or article preparation. Dr Zamani is an employee of Seer Inc (Redwood City, California, USA). All other authors have reported that they have no relationships relevant to the contents of this paper to disclose.

Perspectives **COMPETENCY IN MEDICAL KNOWLEDGE:**

• Circulating proteins mirror key disease mechanisms and serve as potential biomarkers, yet the mouse plasma proteome has remained an underexplored resource in cardiovascular disease research.
• Plasma/serum is dominated by approximately 300 proteins, including apolipoproteins, and complement factors, that mask the presence of potential novel disease-informative biomarkers that span at least a 10^10^-fold dynamic range that is beyond the detection range of mass spectrometers.
• Affinity binding-dependent workflows (using aptamers or antibodies) emerged over a decade ago. They rely on an amplicon or fluorophore readout for protein detection, but limitations of these technologies exist, including the lack of non-human panels, which limits their applicability in animal studies.

**TRANSLATIONAL OUTLOOK:** Recent advances in nanoparticle-dependent protein corona-forming workflows have made plasma proteomics by mass spectrometry feasible. Leveraging a nanoparticle-enrichment strategy previously applied to human plasma, we profiled one of the most widely used mouse models for atherosclerosis. We revealed both known and novel signatures of disease progression, providing the research community with a new resource for biomarker and therapeutic discovery.
