# Supplementary material for: Nanoparticle-Enabled Plasma Proteomics of Experimental Atherosclerosis in Mice
Source: JACC Basic Transl Sci. 2026 Aug 19;11(9):101649. doi: 10.1016/j.jacbts.2026.101649 (PMC13521163; doi:10.1016/j.jacbts.2026.101649)
Supplement: Supplementary file 1 [file mmc2.pdf]

Nanoparticle-enabled plasma proteomics of experimental atherosclerosis in mice

Running title: The *Ldlr-/-* plasma proteome

Constance Delwarde, PhD,[a] Joan T. Matamalas, PhD,[a] Sarvesh Chelvanambi, PhD,[a] Taku Kasai, PhD,[a] Gabriel Shlayen,[a] Diego V. Santinelli-Pestana, MD[a] Maedeh Zamani, MSc, PhD,[b] Elena Aikawa, MD, PhD,[a,c] Masanori Aikawa, MD, PhD,[a,c,d‡] and Sasha A. Singh, MSc, PhD[a,‡]

[a] Center for Interdisciplinary Cardiovascular Sciences, Department of Medicine, Brigham and Women's Hospital, Harvard Medical School, Boston, MA 02115, USA

[b] Seer, Inc. Redwood City, CA 94065, USA

[c] Center for Excellence in Vascular Biology, Department of Medicine, Brigham and Women's Hospital, Harvard Medical School, Boston, MA 02115, USA

[d] Channing Division of Network Medicine, Department of Medicine, Brigham and Women's Hospital and Harvard Medical School, Boston, MA 02115, USA

Supplemental Figures

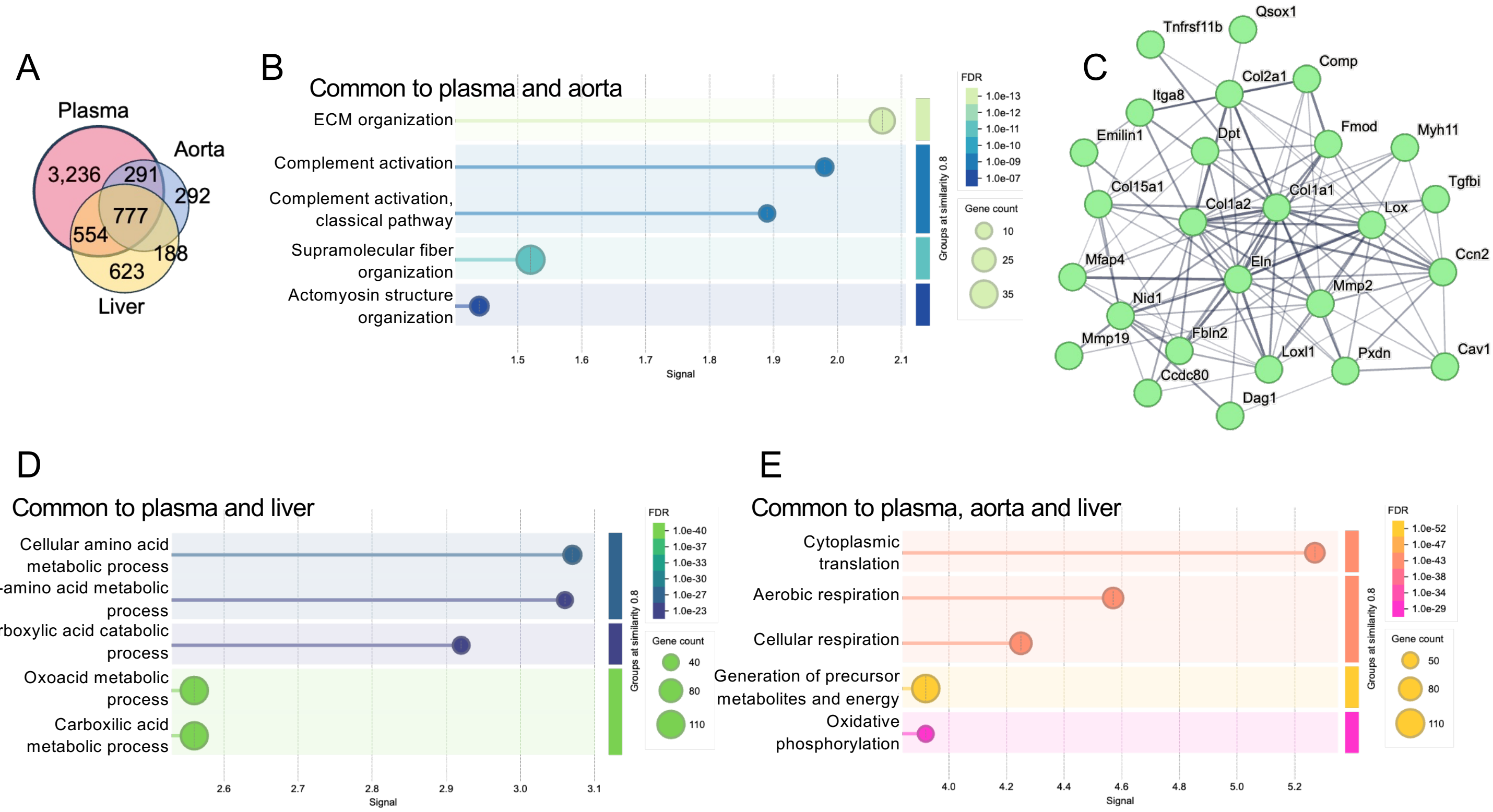

Supplemental Figure 1. Inter-tissue comparisons using the String Database. (A) Venn diagram comparing *Ldlr*$^{-/-}$ plasma, liver, and aorta proteomes. Liver and aorta were acquired using DDA performed on an Orbitrap Exploris 480. **(B)** The genes comprising the top 5 Gene Ontology Biological Process common to plasma and aorta proteomes. **(C)** Protein-protein interaction network for the proteins in ECM organization Biological Process in panel *B*. **(D, E)** The top 5 Biological Processes for the proteins defining plasma and liver overlap **(D)**, and plasma, aorta, and liver overlap **(E)**.

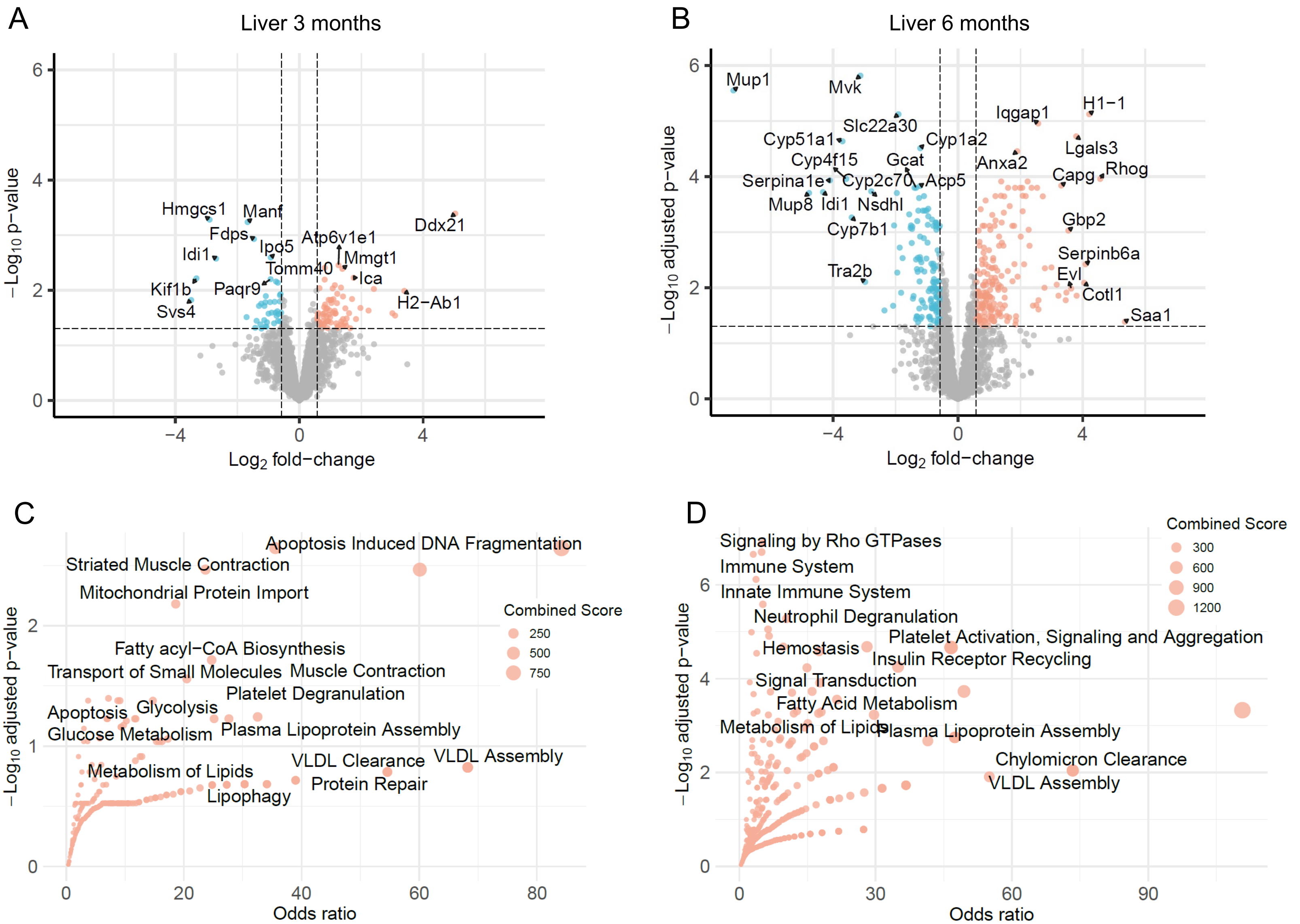

Supplementary Figure 2. HFD-induced changes to the *Ldlr*$^{-/-}$ liver proteome and associated pathways. (A, B) Volcano plots depicting the group mean comparisons (i.e., mean high-fat diet vs mean chow diet) for each 3-month and 6-month timepoint. Orange and blue points indicate proteins with log2 fold-change > 0.58 (orange) or < -0.58 (blue) with a p-value or adjusted p-value < 0.05. n = 6 mice per group. **(C, D)** Reactome enrichment analysis for the high-fat diet-associated increases in the *Ldlr-/-* proteome at 3-month **(C)** and 6-month **(D)**.

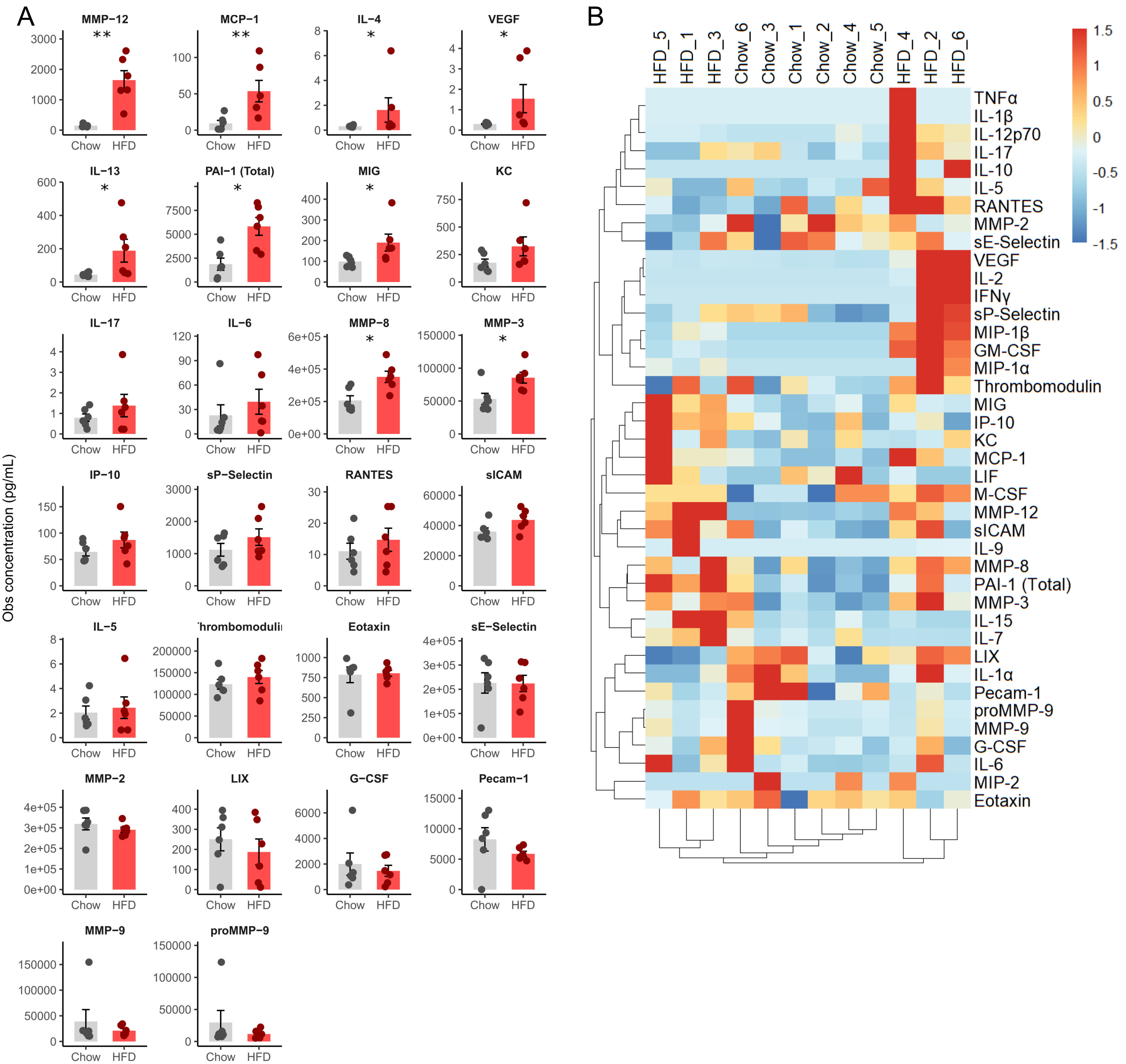

Supplemental Figure 3. ELISA analysis of plasma proteins. (A) Bar plots show plasma protein levels measured by ELISA in six pooled plasma samples per condition collected from 3-month-old mice. Individual data points represent each plasma pool, and bars indicate group means for chow diet *versus* high-fat diet conditions. Statistical comparisons for each protein were conducted using a Mann–Whitney test. Proteins are ordered by effect size. Proteins with more than four out-of-range (OOR) values across samples were excluded from the analysis. Data are presented as individual values with Mean ± SEM. *$p<0.05$ and **$p<0.01$. Grey, chow diet; red, high-fat diet. **(B)** Heatmap showing all protein targets measured across the three ELISA panels. Data are displayed as Z-scores. Values below the limit of detection were imputed using the lower limit of detection (LLOD) for the corresponding protein. Proteins with more than four OOR values across samples were excluded from the analysis.
